# Post-translational modification of RNA m^6^A demethylase ALKBH5 regulates ROS-induced DNA damage response

**DOI:** 10.1093/nar/gkab415

**Published:** 2021-05-28

**Authors:** Fang Yu, Jiangbo Wei, Xiaolong Cui, Chunjie Yu, Wei Ni, Jörg Bungert, Lizi Wu, Chuan He, Zhijian Qian

**Affiliations:** Department of Medicine, UF Health Cancer Center, University of Florida, Gainesville, FL 32610, USA; Department of Biochemistry and Molecular Biology, University of Florida, Gainesville, FL 32610, USA; Department of Chemistry, Department of Biochemistry and Molecular Biology, and Institute for Biophysical Dynamics, The University of Chicago, 929 East 57th Street, Chicago, IL 60637, USA; Howard Hughes Medical Institute, The University of Chicago, 929 East 57th Street, Chicago, IL 60637, USA; Department of Chemistry, Department of Biochemistry and Molecular Biology, and Institute for Biophysical Dynamics, The University of Chicago, 929 East 57th Street, Chicago, IL 60637, USA; Howard Hughes Medical Institute, The University of Chicago, 929 East 57th Street, Chicago, IL 60637, USA; Department of Medicine, UF Health Cancer Center, University of Florida, Gainesville, FL 32610, USA; Department of Molecular Genetics and Microbiology, UF Genetic Institute, University of Florida, FL 32610, USA; Department of Biochemistry and Molecular Biology, University of Florida, Gainesville, FL 32610, USA; Department of Molecular Genetics and Microbiology, UF Genetic Institute, University of Florida, FL 32610, USA; Department of Chemistry, Department of Biochemistry and Molecular Biology, and Institute for Biophysical Dynamics, The University of Chicago, 929 East 57th Street, Chicago, IL 60637, USA; Howard Hughes Medical Institute, The University of Chicago, 929 East 57th Street, Chicago, IL 60637, USA; Department of Medicine, UF Health Cancer Center, University of Florida, Gainesville, FL 32610, USA; Department of Biochemistry and Molecular Biology, University of Florida, Gainesville, FL 32610, USA

## Abstract

Faithful genome integrity maintenance plays an essential role in cell survival. Here, we identify the RNA demethylase ALKBH5 as a key regulator that protects cells from DNA damage and apoptosis during reactive oxygen species (ROS)-induced stress. We find that ROS significantly induces global mRNA *N*^6^-methyladenosine (m^6^A) levels by modulating ALKBH5 post-translational modifications (PTMs), leading to the rapid and efficient induction of thousands of genes involved in a variety of biological processes including DNA damage repair. Mechanistically, ROS promotes ALKBH5 SUMOylation through activating ERK/JNK signaling, leading to inhibition of ALKBH5 m^6^A demethylase activity by blocking substrate accessibility. Moreover, ERK/JNK/ALKBH5-PTMs/m^6^A axis is activated by ROS in hematopoietic stem/progenitor cells (HSPCs) *in vivo* in mice, suggesting a physiological role of this molecular pathway in the maintenance of genome stability in HSPCs. Together, our study uncovers a molecular mechanism involving ALKBH5 PTMs and increased mRNA m^6^A levels that protect genomic integrity of cells in response to ROS.

## INTRODUCTION

Oxidative DNA damage as a result of exposure to reactive oxygen species (ROS) is considered as a major driving force of tumorigenesis that induces chromosomal abnormalities, oncogene activation and promotes genomic instability (1,2). Emerging evidence suggest that gene expression is largely dependent of the state of RNA epigenetic modifications. RNA m^6^A modification has recently been discovered to regulate gene expression through regulation of RNA stability and translation (3,4). However, the role of RNA m^6^A modification in ROS-induced cellular responses has not been investigated in the past.

*N* ^6^-Methyladenosine (m^6^A), the most abundant internal chemical modification of eukaryotic mRNAs, is catalyzed by the m^6^A methyltransferase complex (MTC) which is composed of METTL3, METTL14, WTAP, VIRMA (KIAA1429), RBM15/15B, and ZC3H13 and removed by FTO and ALKBH5 (5–14). m^6^A-sites are recognized by reader proteins including the YT521-B homology (YTH) domain family of proteins (YTHDF1/2/3 and YTHDC1/2) (15–19), the insulin-like growth factor 2 mRNA-binding protein IGF2BPs (IGF2BP1/2/3) (20), heterogeneous nuclear ribonucleoproteins A2/B1 (HNRNPA2B1) (21), proline-rich and coiled-coil-containing protein 2A (PRRC2A) (22), and SND1 (23), which act as functional mediators of m^6^A. m^6^A modification regulates almost every stage of mRNA metabolism including RNA folding as well as mRNA maturation processing, stability, export, and translation (24,25). m^6^A methylation plays an important role in a variety of biological processes by synchronizing expression of hundreds to thousands of mRNAs which facilitates cellular transitions between distinct states during differentiation and development (24). Due to rapid response kinetics, the regulation of mRNA modifications is particularly important under stress conditions (17,26–30). However, it remains unclear which signaling pathways mediate stress-induced RNA m^6^A modifications and how cellular stress regulates m^6^A-modifying proteins.

In this study, we examined the role of RNA demethylase ALKBH5 in response to ROS stress and observed that ROS induces a global increase in mRNA m^6^A via inhibition of the ALKBH5. We determined that ROS inhibits ALKBH5 demethylase activity through ERK/JNK-mediated ALKBH5 phosphorylation at serine residues S87 and S325. ALKBH5 phosphorylation facilitates ALKBH5 SUMOylation by promoting the interaction between ALKBH5 and SUMO E2 UBC9. Notably, ALKBH5 is modified by SUMO-1 mainly at lysine residues K86 and K321, which is mediated by the SUMO E3 ligase PIAS4. Furthermore, we demonstrated that ROS-induced ERK/JNK/ALKBH5 PTMs/m^6^A axis is essential for the maintenance of genome integrity and survival of mammalian cells, and that ERK/JNK/ALKBH5 PTMs/m^6^A axis can be activated in hematopoietic stem/progenitor cells *in vivo* under physiological condition in response endogenous ROS. Collectively, our results demonstrate how the mRNA m^6^A modification adds another dimension to regulation of gene expression of DNA damage repair related genes in response to ROS stress.

## MATERIALS AND METHODS

### Plasmids and antibodies

The pCDH-Strep-ALKBH5 expression plasmid was generated by cloning the corresponding coding sequence into pCDH-Strep vector. All the pCDH-Strep-ALKBH5 K/R (lysine to arginine) or S/A (serine to alanine) mutants were derived from pCDH-Strep-ALKBH5 by site-directed mutagenesis. All expression plasmids for the SUMO systems were kindly provided by Dr. Jiemin Wong's lab. Antibodies used in this study were listed as follows: anti-m^6^A (Synaptic Systems# 202003), anti-γ H2A.X (CST#9719S), anti-γ H2A.X (Thermo Fisher#MA1-2022), anti-ALKBH5 (Sigma#HPA007196), anti-ALKBH5 (Thermo Fisher #703570), anti-FTO (Sigma#SAB2106776), anti-METTL3 (Sigma#SAB2104747), anti-METTL14 (Sigma#HPA038002), anti-SUMO-1 (Thermo Fisher #33–2400), anti-SUMO-2/3 (CST#4971P), anti-ERK (CST#9102S), anti-p-ERK (CST#9106S), anti-JNK (CST#9252S), anti-p-JNK (CST#4671S), anti-phophoserine (Sigma#P5747), anti-phophotyrosine (Sigma#SAB5200015), anti-Strep (Sigma#SAB2702216), anti-Annexin V (Thermo Fisher #17800774), anti-Actin (CST#8457S), anti-Tubulin (CST#2146S), anti-Lamin A/C (CST#4777S), anti-HA (CST#2362), anti-Flag (Sigma#F1804), anti-IGF2BP2 (CST#14672S), anti-eIF3A (CST#3411S).

### Drug treatment

For the ROS-induced DNA damage analysis, the indicated cell lines were treated with or without 100 μM hydrogen peroxide (H_2_O_2_), or 80 μM Carbonyl cyanide m-chlorophenylhydrazone (CCCP) for 6 hours. For the *in vivo* ROS study, DMSO and 5 mg/kg CCCP was intraperitoneally injected in to three pairs of mice. And, all the mice were sacrificed 12 hours after injection.

### Comet assay

Comet assay was performed with the comet kit (R&D SYSTEMS, Cat# 4250-050-K) according to manufactory instructions. Briefly, combine cells at 0.5 million per mLwith molten LMA agarose at a ratio of 1: 10 (v/v) and immediately pipette 50 μl onto comet slice and place it at 4°C for 30 min in the dark. Immerse slice into 4°C lysis buffer for 2 h. Next, immerse slice in alkaline unwinding solution (200 mM NaOH, 1 mM EDTA, pH > 13) for 20 min at room temperature. Finally, Electrophoresis was performed in alkaline electrophoresis solution and the comet slices were stained with SYBR Gold dye. And, the tail length was calculated by image J software.

### Western blot analysis, co-immunoprecipitation and Immunofluorescence staining

The western blot, co-immunoprecipitation and Immunofluorescence staining analyses were performed according to standard protocols as described previously (31), using the indicated antibodies. For examining SUMO-modified proteins, cells were lysed in denaturing buffer (50 mM Tris–HCl pH7.5, 150 mM NaCl, 4% SDS, 1mM EDTA, 8% glycerol, 50mM NaF, 1 mM DTT, 1mM PMSF and protein inhibitors) supplemented with 20 mM *N*-ethylmaleimide (NEM) and heated at 90°C for 10 min. For immunoprecipitation assays, the lysates were further diluted to 0.1% SDS and immunoprecipitated with antibodies against target proteins at 4°C overnight. SUMO-modified proteins were tested by western blotting.

### Streptavidin pull-down analysis

To determine the effect of ALKBH5 SUMOylation on its substrate accessibility, a biotin labeled RNA oligonucleotide bait was synthesized at Ruimian biotechnology, Shanghai, China (5′-biotin-AUGGGCCGUUCAUCUGCUAAAAGG-m^6^A-CUGCUUUUGGGGCUUGU-3′). The pull-down assay was performed according to as described (32). Briefly, transfected HEK293T cells were collected, washed with PBS and lysed in lysis buffer (50 mM Tris–HCl pH 7.5, 150 mM NaCl, 0.5% NP-40, 1 mM EDTA, 8% glycerol supplemented with protease inhibitor mixture, phosphatase inhibitors and 1 mM DTT). 10% of whole cell lysate was used as input and 90% of the whole cell lysate was used for the following Streptavidin sepharose beads pulldown. Next, 2 μg of biotinylated RNA baits were incubated with the above-mentioned whole cell lysate, diluted with binding buffer containing 10 mM Tris–HCl pH 7.5, 150 mM NaCl, 1.5 mM MgCl_2_, 0.05% NP-40 and subjected to rotation at 4°C for 2 h. The resulting beads were washed three times with washing buffer (10 mM Tris–HCl pH 7.5, 150 mM NaCl, 0.05% NP-40, 1 mM EDTA). The effect of SUMOylation on ALKBH5 substrate accessibility was determined by western blotting using anti-ALKBH5 antibodies.

### shRNA knockdown and quantitative RT-PCR

Knockdown of target genes by shRNAs was done as described previously (31). The vector for shRNAs was pLKO.1. The sequences for shRNAs are listed in Supplementary Figure Table S1. For qRT-PCR analysis, total RNA was extracted from various cells as indicated and reverses transcribed using kits purchased from Thermo Fisher. The primer sequences used in the qRT-PCR are listed in Supplementary Figure Table S1.

### Analysis of mRNA m^6^A methylation by dot-blot assay

To analyze mRNA m^6^A methylation, we performed dot-blot assays according to a published procedure with minor changes (33). Briefly, total RNA was extracted using Trizol reagent (Thermo Fisher), and mRNAs were separated using the dynabeads mRNA purification kit (Thermo Fisher). The mRNAs were denatured at 95°C for 5 min, followed by chilling on ice directly. Next, 400 ng mRNAs was spotted to positively charged nylon (GE healthcare), air-dried for 5 min, and cross-linked using a UV cross linker. The membranes were blocked in 5% non-fat milk plus 1% BSA in PBST for 2 hours and then incubated with anti- m^6^A antibodies at 4°C overnight. After three times washing with PBST, the membranes were incubated with Alexa Fluor 680 Goat anti-rabbit IgG secondary antibodies at room temperature for 1 h. Membranes were subsequently scanned using image studio. Methylene blue staining was used as a loading control to make sure equal amount of mRNAs was used for dot-blot analysis.

### mRNA m^6^A methylation quantification by LC-MS/MS

mRNAs from indicated groups were separated by dynabeads mRNA purification kit two times, followed by the removal of contaminated rRNA with RiboMinus™ Eukaryote Kit v2kit (Thermo Fisher). The isolated mRNAs were subsequently digested into nucleotides with nuclease P1 (Sigma, N8630) in 20 ml of buffer containing 25 mM NaCl and 2.5 mM ZnCl_2_ for 1 h at 42°C, followed by 1 unit of FastAP Thermosensitive Alkaline Phosphatase (1 U/μl, Thermofisher Scientific, EF0651) in FastAP buffer were added and the sample was incubated for another 4 h at 37°C. The samples were then filtered (0.22 mm, Millipore) and injected into a C18 reverse phase column coupled online to Agilent 6460 LC–MS/MS spectrometer in positive electrospray ionization mode. The nucleosides were quantified by using retention time and the nucleoside to base ion mass transitions (268-to-136 for A; 282-to-150 for m^6^A. Quantification was performed by comparing with the standard curve obtained from pure nucleoside standards running with the same batch of samples.

### m^6^A-Seq and RNA-Seq analysis

Total RNA was extracted from indicated cells with or without H_2_O_2_ treatment and poly(A)^+^ RNA was further enriched by dynabeads mRNA purification kit (Thermo Fisher). Particularly, DNase I digestion was performed to avoid DNA contamination. mRNA was fragmented by Bioruptor® Pico Sonication System and input was saved before m^6^A IP. m^6^A IP was performed with EpiMark^®^*N*^6^-Methyladenosine Enrichment Kit (NEB, E1610S) following the manufactory protocol. Then, RNA libraries were prepared for both input and IP samples using TruSeq^®^ Stranded mRNA Library Prep (Illumina, 20020594) following the manufactory protocol. Sequencing was performed at the University of Chicago Genomics Facility on an Illumina NextSeq 4000 machine in single-read mode with 50 bp per read at around 25–30 M sequencing depth.

### m^6^A-Seq and RNA-Seq data analysis

Single-end reads were harvested and trimmed by Trim_Galore to remove adaptor sequences and low-quality nucleotides. High-quality reads were then aligned to UCSC hg19 reference genome by HISAT2 using default parameters, and only uniquely mapped reads were retained for all downstream analyses. FeatureCounts software was used to count reads mapped to RefSeq genes, and differentially expressed genes analysis was conducted by Cuffdiff 2 Software. ExomePeak R package was employed to call m^6^A peaks on RefSeq transcripts and further generate differentially methylated m^6^A peaks. Peak centers were then grouped to 3′ UTR, CDS and 5′ UTR by custom scripts. Metagene plots were generated by Guitar package, and motifs were identified by Homer toolkit. To visualize sequencing signals at specific genomic regions, we used Deeptools to normalize all libraries and imported into IGV.

### Biochemistry assay of ALKBH5 activity *in vitro*

Similar to a previous report (34), the demethylation activity assay was performed in standard 20 μL of reaction buffer containing KCl (100 mM), MgCl_2_ (2 mM), SUPERNase In (0.2 U/μl, life technology), L-ascorbic acid (2 mM), α-ketoglutarate (300 μM), (NH_4_)_2_Fe(SO_4_)_2_·6H_2_O (150 μM), and 50 mM of HEPES buffer (pH 6.5). WT and SUMOylation-deficient mutant ALKBH5 was purified from HEK293T cells after the treatment of H_2_O_2_. 100 ng polyadenylated RNA purified from HEK293T cells was incubated with WT or mutant ALKBH5 in the above reaction buffer for 1 hour and then quenched by the addition of 5 mM of EDTA, respectively. Excessive amount of EDTA was added to control samples. The RNAs were then isolated with 100 μL TRIzol^®^ reagents (Thermofisher Scientific, #15596018) using standard protocol and subjected to RNA digestion prior to LC-MS/MS analysis.

### RIP-RT-qPCR

Sixty million cells were collected and re-suspended with RIPA buffer at 4°C for 1 h on a rotator. Then the mRNP lysate was centrifuged at 15 000g for 15 min to clear the lysate. 50 μl cell lysate was saved as input, mixed with 1 ml TRIzol. ALKBH5 antibody or igG was added and incubate at 4°C overnight together with proteinase inhibitor and RNase inhibitor. Cell lysate was then mixed with dynabeads protein A/G (1:1 mixture) with continuously rotating at 4 °C for 4 h. The beads were collected, washed and the binding RNA was extracted by TRIzol reagent. Amount of target transcripts in both the input and IP RNAs were analyzed with RT-qPCR, and IP enrichment ratio of a transcript was calculated as the ratio of its amount in IP to that in the input yielded from same amount of cells and normalized to IgG. The primer sequences used in the qRT-PCR are listed in Supplementary Figure Table S1.

### ROS detection by FACS

ROS detection assay was performed as previously described (35). Briefly, bone marrow cells were stained with cell surface markers as we previously described (36), and were incubated with pre-warmed loading buffer containing the probe DCFHDA (2′-7′-dichlorodihydrofluorescein diacetate) to a final concentration of 5 μM. After 1 h incubation, the intensity of fluorescence was examined by flow cytometry.

### Statistical analysis

Experiments were performed at least three times, and the representative data were shown. All statistical tests were performed using the unpaired Student's test by GraphPad Prism 5 software. A value of *P* < 0.05 was considered statistically significant. In all the results, ‘*’ denotes *P* < 0.05, ‘**’ denotes *P* < 0.01, ‘***’ denotes *P* < 0.001, and ‘ns’ denotes no significant difference.

## RESULTS

### ROS leads to up-regulation of global mRNA m^6^A methylation

**Figure 1.**
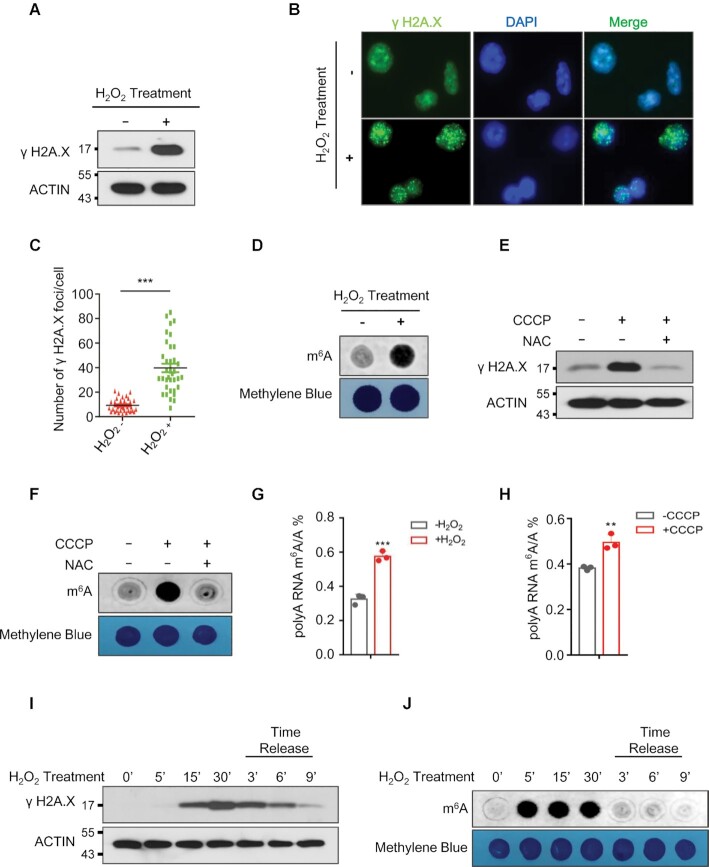
ROS induces up-regulation of global mRNA m^6^A methylation. (**A**, **B**) Western blot (A), and immunostaining (B) analyses showing phosphorylation of H2A.X in HEK293T cells (A), or HeLa cells (B) in the presence or absence of H_2_O_2_. (**C**) Number of γ H2A.X foci derived from the data shown in Figure B. **(D)** Dot-blot assay showing the effect of H_2_O_2_ treatment on global mRNA m^6^A levels in HEK293T cells. (**E**, **F**) Western blot analyses (E) showing phosphorylation of H2A.X, and dot-blot assays (F) were conducted to determine the effect of CCCP treatment on global mRNA m^6^A levels in HEK293T cells with or without N-acetyl-L-cysteine (NAC) treatment. (**G**, **H**) LC–MS/MS analyses indicating ROS stress induced global mRNA m^6^A modification. (**I**, **J**) Western blot analysis (I), and dot-blot analysis (J) showing the kinetics of H2A.X phosphorylation and global mRNA m^6^A methylation in the presence, or absence of H_2_O_2_ treatment respectively.

To examine the role of mRNA m^6^A methylation in response to ROS stress, we treated human cell lines with H_2_O_2_. Consistent with prior studies (37), H_2_O_2_ induced DNA damage in HEK293T and HeLa cells as evidenced by increased expression of phosphorylated H_2_AX (γH_2_AX), a sensitive marker of DNA damage and genomic instability (38,39) (Figure 1A–C). Furthermore, H_2_O_2_ significantly increased global mRNA m^6^A methylation in human HEK293T and HeLa cells (Figure 1D and Supplementary Figure S1A). Carbonyl cyanide m-chlorophenylhydrazone (CCCP) was used to mimic endogenous ROS activation under physiological conditions (40–43). Similar to H_2_O_2,_ CCCP treatment significantly induced phosphorylation of H_2_AX as well as global mRNA m^6^A levels in human HEK293T (Supplementary Figure S1B and C) and HeLa cells (Supplementary Figure S1D and E). More importantly, CCCP-induced DNA damage and increase in global mRNA m^6^A methylation was inhibited by *N*-acetyl-l-cysteine (NAC), which abrogates ROS generated by CCCP treatment, suggesting that CCCP-induced DNA damage and global increase in global mRNA m^6^A methylation was mainly attributed to CCCP-induced ROS (Figure 1E and F). We next performed quantitative analysis of the mRNA m^6^A/A ratio by LC–QqQ–MS/MS using previously described protocol (44). Consistent with the dot blot analysis, both H_2_O_2_- and CCCP-induced ROS significantly increased global mRNA m^6^A levels (Figure 1G and H). To determine the effect of ROS treatment on kinetics of DNA damage and global mRNA m^6^A methylation, we treated 293T cells with H_2_O_2_ at different time intervals and performed H_2_O_2_ time release analyses. As shown in Figure 1I, phosphorylation of H2A.X was significantly induced 15 min after H_2_O_2_ treatment, peaking at 30 minutes, and diminishing over the following 9 minutes after H_2_O_2_ release. Compared with DNA damage, induction of global mRNA m^6^A methylation comes first, which occurred at 5 min after H_2_O_2_ treatment and dropped dramatically after H_2_O_2_ release (Figure 1J). Together, these data indicate that exogenous or endogenous ROS-induced stress significantly up-regulates global mRNA m^6^A modification.

### ROS promotes mRNA m^6^A demethylase ALKBH5 SUMOylation

To determine how H_2_O_2_ induces mRNA m^6^A methylation, we examined the expression levels of key mRNA m^6^A methylation writers including METTL3 and METTL14, and mRNA m^6^A erasers FTO and ALKBH5 by RT-qPCR and western blot analysis. As shown in Supplementary Figure S2A–C, H_2_O_2_-induced ROS had no effect on transcription or protein levels of mRNA m^6^A erasers, whereas it significantly increased both the transcript and protein levels of mRNA m^6^A writers (Supplementary Figure S2C–E). This suggests that ROS-induced mRNA m^6^A modification may occur through up-regulation of m^6^A writers METTL3 and METTL14. However, METTL3 or METTL14 knockdown by specific shRNAs (Supplementary Figure S3A and S3B) only partially blocked ROS-induced up-regulation of global mRNA m^6^A modification (Supplementary Figure S3C and S3D), suggesting the presence of an additional molecular mechanism that mediates the ROS-induced increase of global mRNA m^6^A modification.

**Figure 2.**
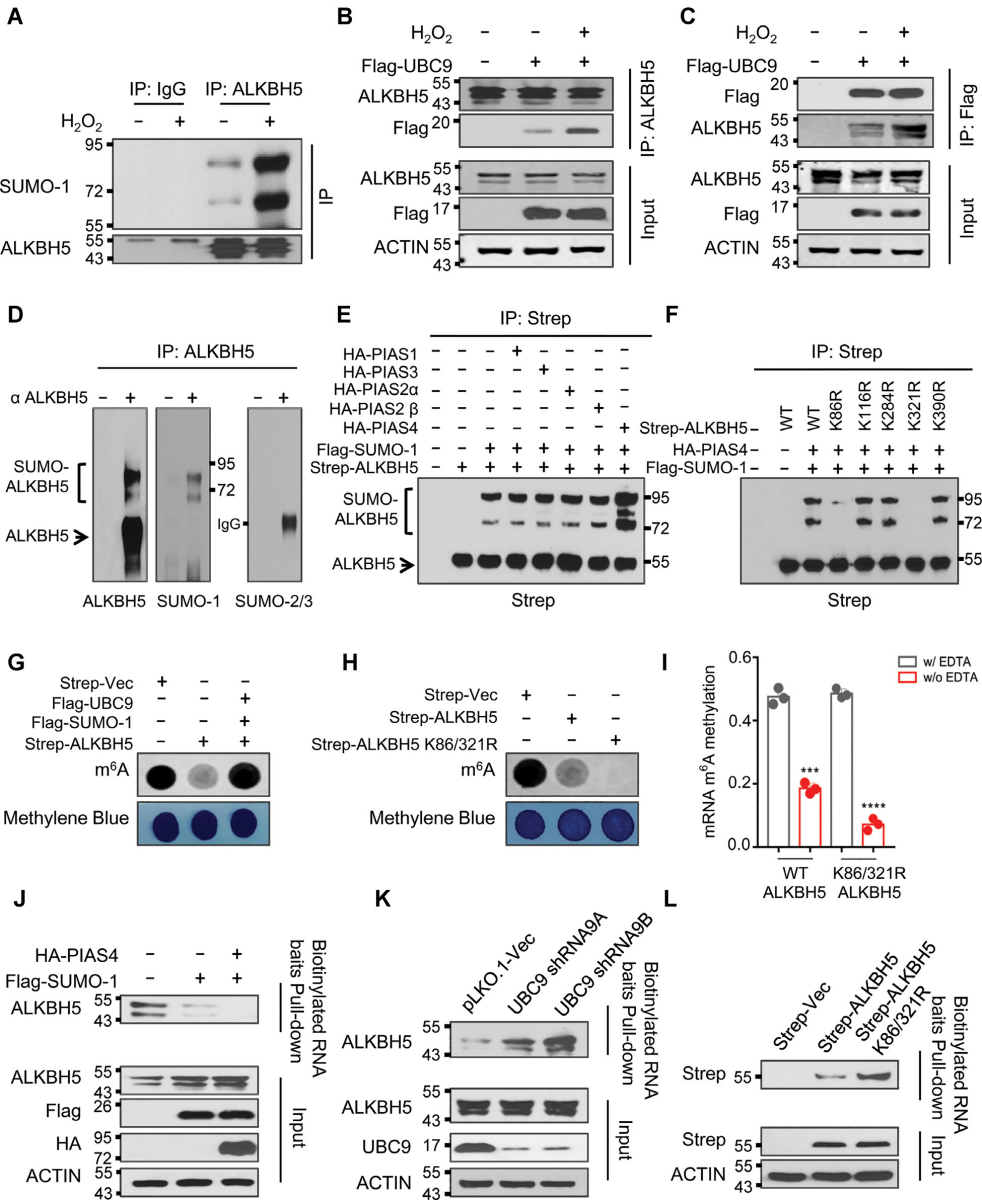
ROS induces mRNA m^6^A demethylase ALKBH5 SUMOylation. (**A**) Denaturing immunoprecipitation (IP) assay indicating H_2_O_2_-induced ROS selectively enhances ALKBH5 SUMOylation. (**B**, **C**) Reciprocal IP analyses showing ROS facilitates the interaction between ALKBH5 and SUMO E2 conjugation enzyme UBC9. Reciprocal IP assays were performed in cells ectopically expressing Flag-UBC9 with or without H_2_O_2_ treatment. IP antibodies are shown on the right and western blotting antibodies are shown on the left. (**D**) Denaturing IP analyses showing that endogenous ALKBH5 is modified by SUMO-1 but not by SUMO-2/3 in HEK293T cells. (**E**) Denaturing IP analyses showing that SUMO E3 ligase PIAS4 mediates ALKBH5 SUMOylation. HEK293T cells were transfected with or without Strep-ALKBH5, HA tagged PIAS family SUMO E3 ligases and Flag-SUMO-1. The cells were collected for denaturing IP analyses two days after transfection. (**F**) Denaturing IP analyses showing ALKBH5 SUMOylation mainly occurs at lysine residues K86 and K321. Strep tagged wild-type ALKBH5 or ALKBH5 lysine (K) to arginine (R) mutants were solely expressed or co-expressed with HA-tagged PIAS4 and Flag-SUMO-1 in HEK293T cells. (**G**) Dot-blot analyses showing ALKBH5 mRNA m^6^A demethylase activity is inhibited by SUMOylation. Strep-tagged ALKBH5 was expressed alone or co-expressed with Flag tagged UBC9 and SUMO-1 in HEK293T cells. (**H**) Dot-blot assays indicating that blocking ALKBH5 SUMOylation markedly facilitates ALKBH5 mRNA m^6^A demethylase activity. Strep tagged vector, or vectors expressing wild-type ALKBH5 or SUMOylation-deficient mutant ALKBH5 (ALKBH5 K86/321R) were transfected into HEK293T cells. 48 hours after transfection, dot-blot analyses were conducted to detect global mRNA m^6^A levels. (**I**) *In vitro* mRNA m^6^A demethylase activity analyses indicating that blocking ALKBH5 SUMOylation dramatically facilitates ALKBH5 mRNA m^6^A demethylase activity. Strep tagged wild-type or SD- mutant ALKBH5 (ALKBH5 K86/321R) were expressed in HEK293T cells. (**J–L**) Substrate pull-down analyses showing that ALKBH5 SUMOylation dramatically blocks its substrate accessibility. Flag tagged SUMO-1 was expressed alone or co-expressed with HA-tagged PIAS4 in HEK293T cells (J). sh-vector, or two vectors expressing shRNAs against UBC9 were transfected into HEK293T cells (K). Strep tagged wild-type or SD-mutant ALKBH5 was expressed in HEK293T cells. (L). Forty-eight hours after transection, whole cell lysates were subjected to biotin labeled m^6^A-containing RNA oligo pull-down.

Since SUMOylation regulates a variety of cellular processes including cellular response to DNA damage (45), we evaluated the possibility that ROS regulates global mRNA m^6^A methylation via SUMOylation of mRNA m^6^A modification writers or erasers. We found that ROS specifically promotes ALKBH5 SUMOylation (Figure 2A and Supplementary Figure S4) but not FTO, METTL3 and METTL14 (Supplementary Figure S5A–C). We have also observed SUMOylation of METTL3 in HEK293T cells (Supplementary Figure S5B), consistent with previously published data (33). However, ROS did not induce METTL3 SUMOylation. Furthermore, ALKBH5 SUMOylation could also be promoted by CCCP-induced endogenous ROS (Supplementary Figure S5D). Since E2 conjugation enzyme UBC9 is critical for mediating protein SUMOylation (46–48), we explored the effects of ALKBH5 SUMOylation on its interaction with UBC9. Co-immunoprecipitation analyses showed that ROS strongly increases the interaction between ALKBH5 and UBC9 (Figure 2B and C). In addition, numerous studies also suggest that Sentrin/SUMO-specific proteases 1 and 3 (SENP1 and SENP3) are involved in regulation of protein SUMOylation under oxidative stress (49–51). Therefore, we next determined the effect of ROS on the interaction between ALKBH5 and SENP1. Consistent with previously published results (50,51), SENP3 was stabilized by H_2_O_2_ treatment in 293T cells. However, we did not observe the interaction between ALKBH5 and SENP3. (Supplementary Figure S6). Interestingly, the interaction between ALKBH5 and SENP1 was significantly disrupted by H_2_O_2_ treatment (Supplementary Figure S6). Together, these data provide evidence that ROS specifically promotes ALKBH5 but not FTO, METTL3 and METTL14 SUMOylation by enhancing the interaction of ALKBH5 and UBC9 and inhibiting the association between ALKBH5 and SENP1.

### ALKBH5 SUMOylation blocks m^6^A demethylase activity by inhibition of substrate accessibility

There are three SUMO proteins including SUMO-1, SUMO-2 and SUMO-3, which can be covalently conjugated to the targeted proteins (52). We next determined whether ectopically expressed ALKBH5 could be SUMOylated by SUMO-1, SUMO-2 and/or SUMO-3. Immunoblotting of the ALKBH5 immunoprecipitates using strep-tactin beads identified high apparent molecular weight ALKBH5 species in HEK293T cells with co-expression of ALKBH5 and SUMO-1 but not in cells co-expression of SUMO-2 or SUMO-3 (Supplementary Figure S7A–C), indicating that ALKBH5 is conjugated with SUMO-1 but not SUMO-2/3. In addition to mono-SUMOylated ALKBH5, higher molecular weight ALKBH5 species were also detected, suggesting that ALKBH5 is modified with SUMO-1 at multiple lysine residues or SUMO-1 is linked to ALKBH5 in polymer chain. Consistent with the observation for exogenous Strep-ALKBH5 in HEK293T cells, immunoprecipitation of the endogenous ALKBH5 from 293T cells showed the presence of SUMO-1 high molecular weight species but not SUMO-2/3 modified forms (Figure 2D and Supplementary Figure S8B).

In eukaryotes, there are a number of SUMO E3 ligases, which increase the efficiency of SUMO conjugation and accelerate the rate of SUMO modification (53). The largest class of SUMO E3 ligase is the PIAS family proteins, which includes PIAS1, PIAS2 PIAS3, and PIAS4, that all share a RING domain (54–58). To examine the mechanism underlying ALKBH5 SUMOylation, we co-expressed PIAS family proteins with ALKBH5 and SUMO-1, and performed denaturing IP to delineate which SUMO E3 ligase mediates ALKBH5 SUMOylation. This analysis showed that among all PIAS family proteins, only co-expression of ALKBH5 with PIAS4 and SUMO-1 resulted in extensive SUMOylation of ALKBH5 (Figure 2E, last lane and Supplementary Figure S8C). Additionally, co-immunoprecipitation assay revealed that ALKBH5 interacts with PIAS4 (Supplementary Figure S8A).

SUMOylation of substrates frequently occurs at a lysine within the canonical SUMOylation consensus motif ψKx (D/E), in which ψ represents a large hydrophobic residue and x represents any amino acid followed by an acidic residue (59). By using two independent algorithms GPS-SUMO (60) and JASSA (61), we identified five potential SUMOylation sites in ALKBH5, which included K390, K86, K321, K284 and K116 (Supplementary Figure S9A). We generated ALKBH5 SUMOylation-deficient mutants with lysine replaced by arginine, a charged aliphatic acid, within the putative SUMOylation motifs. We performed denaturing IP analyses to determine which lysine mutation abolished ALKBH5 SUMOylation. As shown in Figure 2F and Supplementary Figure S9B, ALKBH5 K86R mutation reduced ALKBH5 SUMOylation dramatically, and ALKBH5 K321R mutation nearly abolished ALKBH5 SUMOylation. In addition, we also established that ALKBH5 K86R/K321R double mutant is not SUMOylated (Supplementary Figure S10), suggesting that K86 and K321 are the major ALKBH5 SUMOylation sites. Notably, ALKBH5 overexpression significantly reduced the level of mRNA m^6^A methylation while co-expression of ALKBH5, UBC9 and SUMO-1, promoting ALKBH5 SUMOylation, did not affect m^6^A methylation levels (Figure 2G and Supplementary Figure S11). Furthermore, the ALKBH5 SUMOylation-deficient mutant (ALKBH5 K86R/K321R) inhibited global mRNA m^6^A methylation more efficiently than wild type ALKBH5 (Figure 2H and Supplementary Figure S12). *In vitro* assays revealed that the ALKBH5 K86R/K321R mutant protein isolated from HEK293T cells had a significantly higher mRNA m^6^A demethylation activity than wild-type ALKBH5 (Figure 2I). Taken together, the data demonstrate that SUMOylation of ALKBH5 inhibits its m^6^A demethylase activity *in vivo* and *in vitro*.

Next, we analyzed how ALKBH5 SUMOylation regulates its m^6^A demethylase activity. Western blot analysis showed that increasing ALKBH5 SUMOylation by PIAS4 or SUMO-1 overexpression or reducing ALKBH5 SUMOylation by UBC9 knockdown, does not alter ALKBH5 protein levels (Supplementary Figure S13A and B). Additionally, increasing ALKBH5 SUMOylation by overexpression of PIAS4 and SUMO-1 did not affect ALKBH5 subcellular localization (Supplementary Figure S13C). Furthermore, wild type and SUMOylation-deficient mutant ALKBH5 had the same subcellular localization (Supplementary Figure S13D). Consistently, reducing endogenous ALKBH5 SUMOylation by UBC9 knockdown did not affect its subcellular localization (Supplementary Figure S13E). Next, we performed a substrate pull-down assay using synthesized biotinylated m^6^A-containing RNA baits to capture endogenous ALKBH5 from the HEK293T cells expressing vector, SUMO-1, or SUMO-1 plus PIAS4. The result showed that SUMO-1 overexpression markedly inhibits the binding ability of ALKBH5 to its substrate, and that the inhibitory effect of SUMO-1 on ALKBH5 substrate binding activity was further augmented by co-expression of PIAS4 and SUMO-1 (Figure 2J). In contrast, suppression of ALKBH5 SUMOylation by knocking down UBC9 enhanced ALKBH5 substrate binding activity (Figure 2K). In addition, we found that the ALKBH5 K86R/K321R double mutant significantly enhanced ALKBH5 substrate binding activity (Figure 2L). Collectively, our results show that SUMO E3 ligase PIAS4 mediates ALKBH5 SUMOylation at lysine residues K86 and K321, and that ALKBH5 SUMOylation markedly inhibits its mRNA m^6^A demethylase activity by blocking its ability to bind to m^6^A RNA species.

### SUMOylation-deficient ALKBH5 overexpression blocks ROS-induced mRNA m^6^A methylation, leading to a significant delay of DNA repair and increase of cell apoptosis

**Figure 3.**
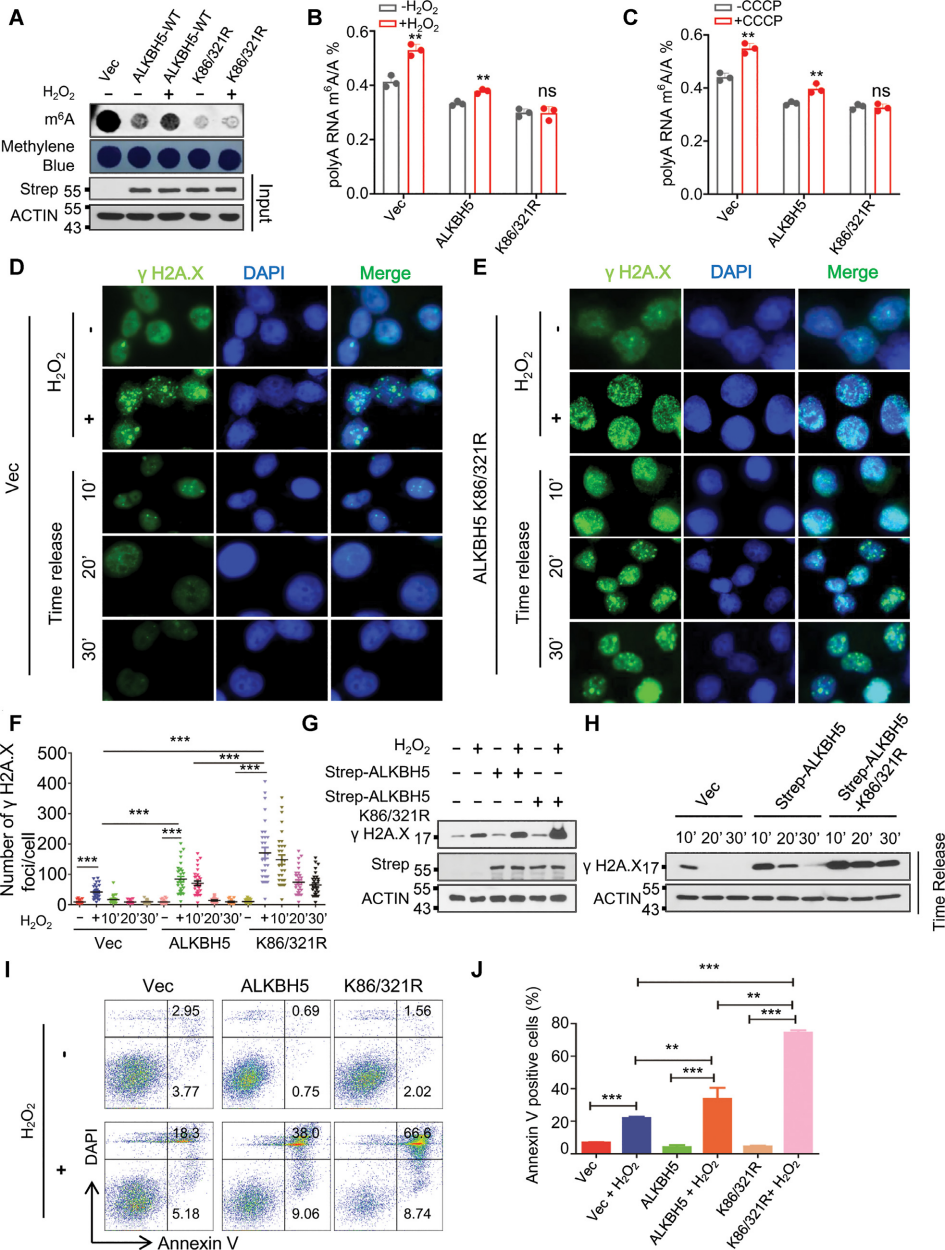
ALKBH5 SUMOylation is mainly responsible for ROS-induced global increase in mRNA m^6^A methylation. (**A**) Dot-blot assay showing that SUMOylation-deficient mutant ALKBH5 overexpression completely blocks ROS-induced global increase in mRNA m^6^A methylation. Strep tagged wild-type or SD-mutant ALKBH5 was transfected into HEK293T cells. Half of cells were used for western blot analysis to confirm the expressions of ectopically expressed plasmids, and the rest of cells were used for dot-blot analysis. (**B**, **C**) LC–MS/MS analyses indicating ROS induces global mRNA m^6^A methylation via ALKBH5 SUMOylation. HEK293T cells stably expressing vector, wild-type, or SUMOylation-deficient mutant ALKBH5 were treated with or without H_2_O_2_ (B) or CCCP (C) for 6 h, and subjected to LC–MS/MS analysis of mRNA m^6^A methylation. (**D, E**) γ H2A.X immunostaining analysis showing that SUMOylation-deficient mutant ALKBH5 overexpression dramatically delays H_2_O_2_–induced DNA damage repair. HeLa cells stably expressing Vec (D), or ALKBH5 K86/321R (E) were treated with H_2_O_2,_ which was removed after 6 h. γ H2A.X immunostaining analyses were performed at time intervals between 10 and 30 min. (**F**) γ H2A.X foci quantitative data for D, E and Supplementary Figure S17. (**G**, **H**) Western blot analysis showing that SUMOylation-deficient mutant ALKBH5 overexpression significantly inhibits H_2_O_2_–induced DNA damage repair. (**I**) Cell apoptosis analyses showing ALKBH5 K86/321R overexpression markedly increases ROS-induced cell apoptosis. Apoptosis analyses were performed in HEK293T cells transfected with Strep tagged vector, or vectors expressing ALKBH5 or Strep tagged ALKBH5 K86/321R in the presence or absence of H_2_O_2_. (**J**) Histograms showing the summary and statistical analysis of Figure 3I.

To determine whether ALKBH5 SUMOylation plays a vital role in ROS-induced global mRNA m^6^A modification, we ectopically expressed wild-type or SUMOylation-deficient mutant ALKBH5 in HEK293T cells (Figure 3A) and treated the cells with H_2_O_2_. As shown in Figure 3A, ROS-induced global mRNA m^6^A modification was partially reduced by wild-type ALKBH5 overexpression, whereas it was completely blocked by overexpression of ALKBH5 K86R/K321R mutant. Depletion of ALKBH5 by CRISPR-Cas9-mediated deletion in HEK293T cells was confirmed by western blot (Supplementary Figure S14A), T7E1 digestion (Supplementary Figure S14B), and DNA sequencing (Supplementary Figure S14C), and ALKBH5 knockout remarkably increased global mRNA m^6^A modification (Supplementary Figure S14D). We employed LC–QqQ–MS/MS analysis to establish m^6^A/A ratio under peroxide and CCCP-induced conditions. We observed that both H_2_O_2_- and CCCP-induced ROS significantly increased mRNA m^6^A modification in 293T cells stably expressing vector, or wild-type ALKBH5, but did not induce mRNA m^6^A methylation levels in ALKBH5 knockout or ALKBH5 K86/K321R mutant-expressing cells (Figure 3B, C and Supplementary Figure S15A-15B), suggesting that ROS induces an increase in global mRNA m^6^A methylation mainly through SUMO modification of ALKBH5.

To determine the effect of inhibition of ROS-induced m^6^A methylation by SUMOylation-deficient mutant ALKBH5 on ROS-induced DNA damage, we performed comet analysis (single cell gel electrophoresis assay), which is used for quantitating DNA damage and repair with single cell resolution (62,63). As shown in Supplementary Figure S16A and B, ALKBH5 overexpression significantly promoted H_2_O_2_- induced DNA damage, and SUMOylation-deficient mutant ALKBH5 caused more DNA damage than wild-type ALKBH5. We next performed γ H2A.X immunostaining analysis in HeLa cells stably expressing Vector, wild-type, or SUMOylation-deficient mutant ALKBH5. As indicated by γ H2A.X foci, H_2_O_2_-induced DNA lesions were repaired within 10 minutes and 20 minutes after removal of H_2_O_2_ in Vector and wild-type ALKBH5 expressing cells respectively (Figure 3D, F and Supplementary Figure S17), whereas damaged DNA still persisted in 30 minutes after removal of H_2_O_2_ in cells expressing the SUMOylation-deficient mutant ALKBH5 (Figure 3E and F), indicating that ROS-induced DNA damage repair can be markedly delayed in the presence of SUMOylation-deficient mutant ALKBH5. Similar results have been observed by Western blot analysis (Figure 3G and H). In addition, we showed that both wild-type ALKBH5 and ALKBH5 K86R/K321R mutant overexpression had no effect on cell survival in the absence of ROS stress, but significantly sensitized HEK293T cells to ROS (Figure 3I and 3J). Of note, cells expressing ALKBH5 SUMOylation-deficient mutant had a much higher frequency of apoptosis than the wild-type ALKBH5 overexpressing cells (Figure 3I and J). Thus, the data show that ALKBH5 SUMOylation plays a crucial role in ROS-induced global mRNA m^6^A modification, DNA damage repair and cell survival.

### ROS induces ALKBH5 phosphorylation and SUMOylation by activation of ERK/JNK signaling

**Figure 4.**
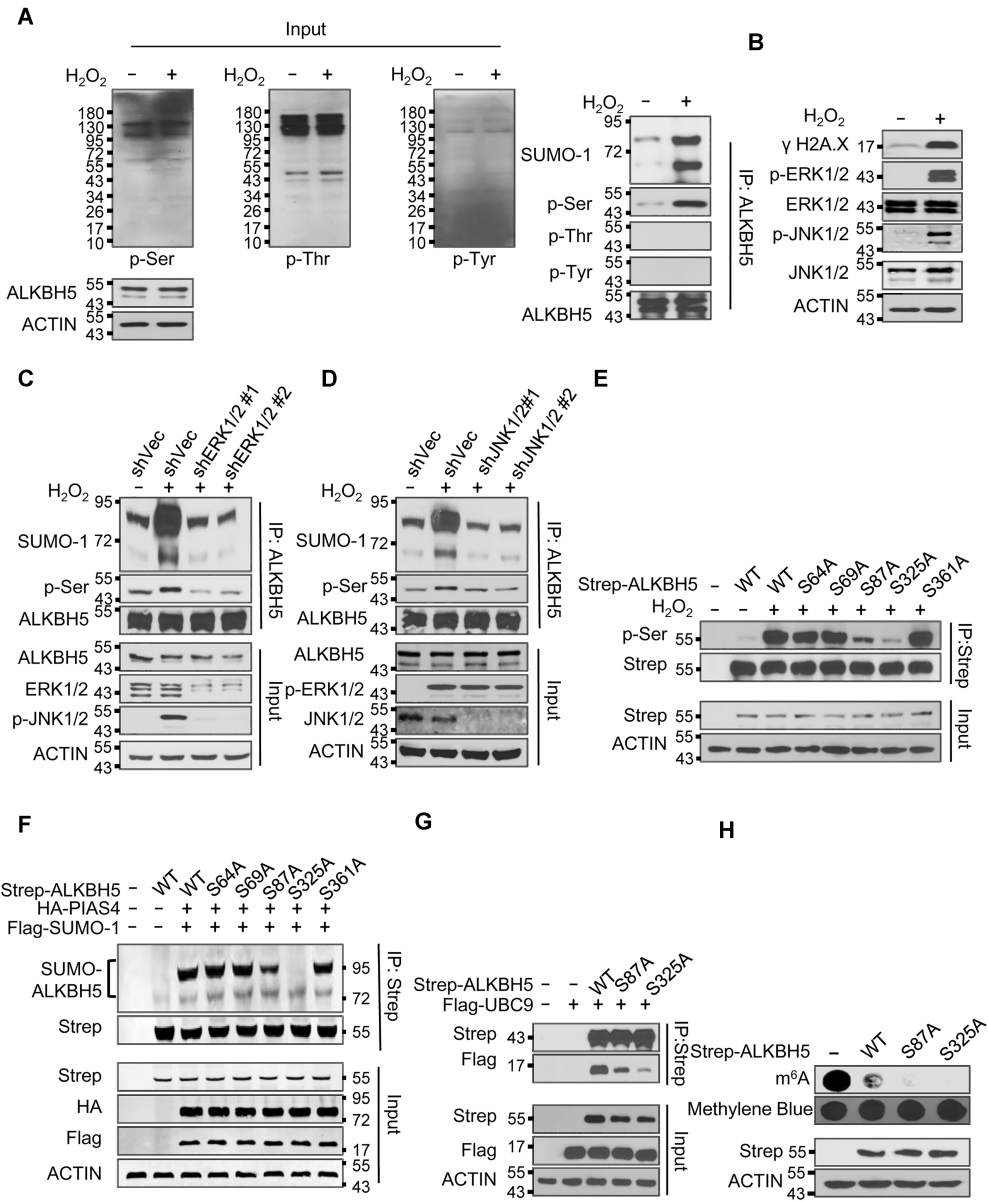
ROS induces ALKBH5 phosphorylation and SUMOylation by activating ERK/JNK signaling. (**A**) Denaturing IP analysis indicating ROS significantly induces ALKBH5 phosphorylation and SUMOylation in HEK293T cells. (**B**) Western blot analysis indicating ROS dramatically activates the ERK/JNK signaling pathway in HEK293T cells. (**C**) Denaturing IP assay indicating inhibition of ERK dramatically blocks ROS-induced ALKBH5 phosphorylation and SUMOylation. (**D**) Denaturing IP analysis suggesting inhibition of JNK significantly inhibits ROS-induced ALKBH5 phosphorylation and SUMOylation. (**E**) IP analysis showing that ROS induces ALKBH5 phosphorylation at serine residues S87 and S325. (**F**) Denaturing IP analysis indicating that blocking ALKBH5 phosphorylation markedly inhibits ALKBH5 SUMOylation. Strep-tagged wild-type ALKBH5 or phosphorylation-deficient ALKBH5 was overexpressed alone or co-expressed with HA-tagged PIAS4 and Flag-tagged SUMO-1 in HEK293T cells. Denaturing IP assays were performed to determine the effect of blocking ALKBH5 phosphorylation on ALKBH5 SUMOylation. (**G**) Co**-**IP analysis showing that blocking ALKBH5 phosphorylation inhibits the interaction between ALKBH5 and SUMO E2 UBC9. Flag-tagged UBC9 was overexpressed alone or co-expressed with Strep tagged wild-type or phosphorylation-deficient ALKBH5 in HEK293T cells and Co**-**IP assays were performed to determine the effect of blocking ALKBH5 phosphorylation on the interaction between ALKBH5 and UBC9. **(H)** Dot-blot analysis indicating that blocking ALKBH5 phosphorylation dramatically increases ALKBH5 mRNA m^6^A demethylase activity. Strep tagged wild-type or phosphorylation-deficient ALKBH5 was expressed in HEK293T cells. Two days after transfection, the cells were collected and subjected to dot-blot analysis.

Phosphorylation–dependent SUMOylation modifications were described previously (64). Thus, we aimed to determine whether ROS induced ALKBH5 phosphorylation. As shown in Figure 4A and Supplementary Figure S18A, both H_2_O_2_ and CCCP treatment induced ALKBH5 serine but not tyrosine phosphorylation. Threonine phosphorylation of ALKBH5 was not induced by H_2_O_2_ either (Figure 4A). It has been reported that the mitogen-activated protein kinase (MAPK) signaling pathway, which includes the extracellular regulated protein kinase 1/2 (ERK1/2), c-Jun N-terminal kinase (JNK) and p38 regulatory pathways, is activated by ROS stress (65,66). We thus investigated whether ERK/JNK signaling pathways mediated ROS-induced ALKBH5 phosphorylation and SUMOylation. Both H_2_O_2_ and CCCP induced DNA damage and activated the ERK/JNK signaling pathway (Figure 4B and Supplementary Figure S18B). ERK1/2 knockdown by ERK1/2 specific shRNAs suppressed ROS-induced activation of JNK (Figure 4C), suggesting that JNK is a downstream mediator of ERK1/2 in response to ROS. Denaturing IP assays revealed that ROS-induced ALKBH5 phosphorylation and SUMOylation were inhibited by either ERK or JNK knockdown (Figure 4C and D), suggesting that activation of ERK, followed by activation of JNK are necessary for ROS-induced ALKBH5 phosphorylation and SUMOylation. More importantly, the interaction between ALKBH5 and JNK1/2 was significantly increased by H_2_O_2_ treatment (Supplementary Figure S19).

Previous studies showed that substrate SUMOylation is facilitated by phosphorylation of the upstream or downstream serine, tyrosine and threonine sites of the substrate (67,68). We showed that H_2_O_2_ treatment induced serine but not threonine phosphorylation of ALKBH5 (Figure 4A). To identify the serine sites that are phosphorylated by ROS-activated JNK signaling, we mutated five serine residues, S64, S69, S87, S325 and S361, to alanine. These serine residues are close to the identified SUMOylation sites (lysines K86 and K321) of ALKBH5 wild-type. Thus, wild-type or distinct serine to alanine mutants of ALKBH5 were expressed in HK293T cells for further analysis. As shown in Figure 4E, ROS-induced ALKBH5 phosphorylation was significantly reduced by the ALKBH5 S87A mutation but not the S64A, S69A or S361A mutations. Phosphorylation was almost completely blocked by the S325A mutation, suggesting that S87 and S325 are critical ROS-sensitive ALKBH5 phosphorylation sites. Additionally, denaturing IP showed that the ALKBH5 S325A mutation completely abrogates ALKBH5 SUMOylation, while the ALKBH5 S87A, but not the S64A, S69A and S361A mutations, significantly inhibit ALKBH5 SUMOylation (Figure 4F), confirming that ALKBH5 phosphorylation at serine 87 and serine 325 promotes ALKBH5 SUMOylation in response to ROS stress. Co-IP assay showed that the ALKBH5 S87A mutation inhibited the interaction between ALKBH5 and UBC9, and that the ALKBH5 S325A mutant had a stronger inhibitory effect on the interaction between ALKBH5 and UBC9 compared to the ALKBH5 S87A mutant (Figure 4G). Meanwhile, the interaction between ALKBH5 and SENP1 was significantly enhanced by ALKBH5 S87A and S325A mutants (Supplementary Figure S20). In addition, we showed that ALKBH5 S325A possessed the strongest mRNA m^6^A demethylase activity while ALKBH5 S87A had a higher demethylase activity than wild-type ALKBH5 (Figure 4H). Collectively, these results suggest that ROS induces ALKBH5 phosphorylation at serine 87 and serine 325 by activation of ERK/JNK signaling, and that ALKBH5 phosphorylation promotes ALKBH5 SUMOylation not only by facilitating the interaction between ALKBH5 and UBC9, but also by inhibiting the association between ALKBH5 and SENP1 at the same time.

### Global gene expression profiling identifies DNA damage repair genes as ALKBH5 downstream targets induced by ROS

**Figure 5.**
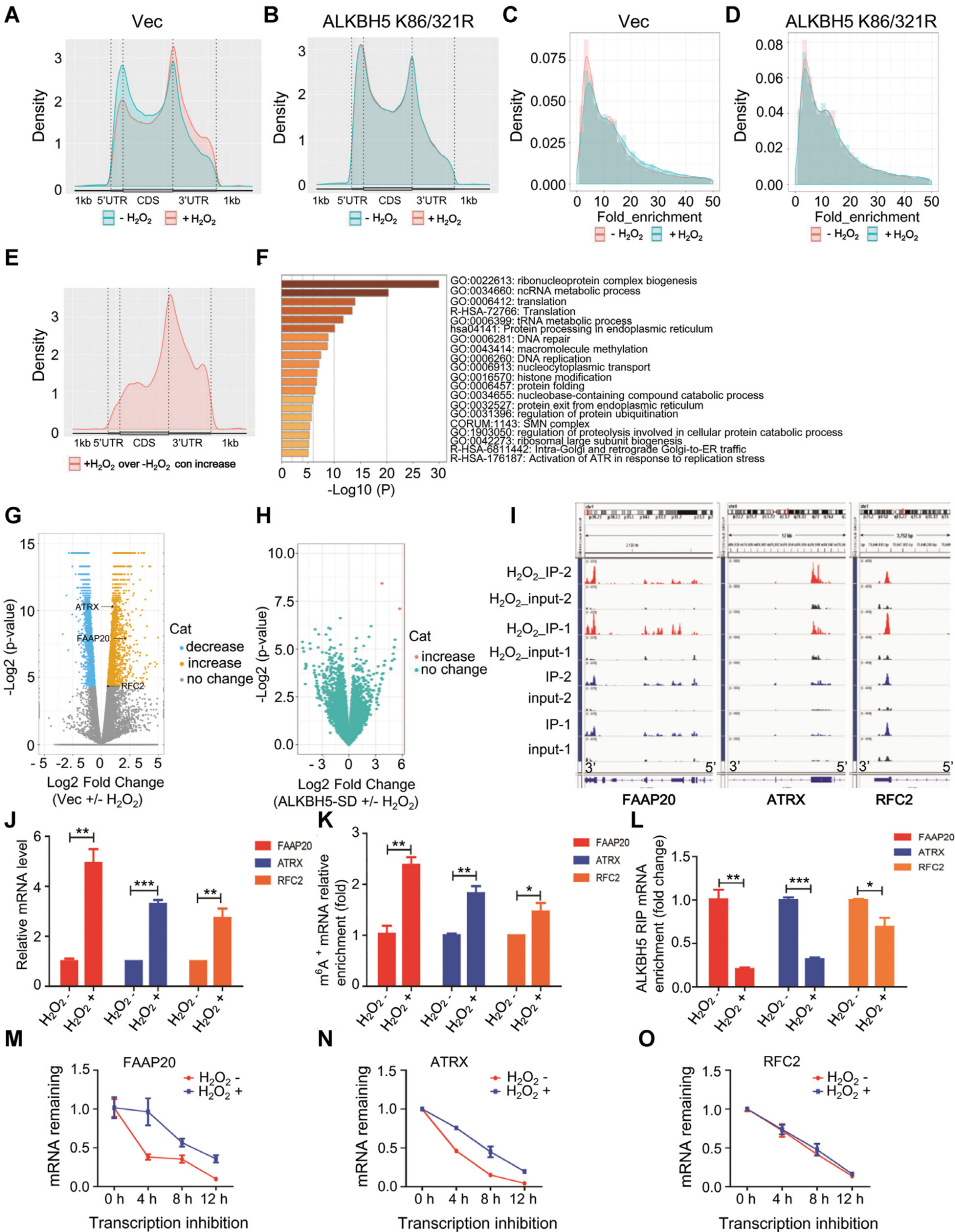
Global gene expression profiling indicates ALKBH5 downstream targets related to DNA damage repair are induced by ROS. (**A**, **B**) The frequency distribution of m^6^A peaks across the length of mRNA transcripts shown by metagene in control cells (A) or in cells expressing ALKBH5 K86/321R (B) with or without H_2_O_2_ treatment. Each region of the 5′ untranslated region (5′ UTR), coding region (CDS), and 3′ untranslated region (3′ UTR) was split into 100 segments, and the percentage of m^6^A peaks that fall within each segment was determined. (**C**, **D**) The density (line) and frequency (histogram) of m^6^A peaks in control samples (C) or SD-mutant overexpressed samples (D) with or without H_2_O_2_ treatment. (**E**) The adjusted density (line, top) and distribution (histogram, bottom) of hyper peaks from (C) across different mRNA regions in control samples with or without H_2_O_2_ treatment. (**F**) Enrichment analysis for significantly increased peaks of m^6^A modification from (C) in control samples with or without H_2_O_2_ treatment. (**G**) Differentially expressed genes shown in volcano figure in control samples with or without H_2_O_2_ treatment. There were 1051 genes withy significantly reduced expression (log_2_FC < 0, *P* < 0.01), 1106 genes with significantly increased expression (log_2_FC > 0, *P* < 0.01) and 15 102 genes without statistically significant changes in expression. (**H**) Differentially expressed genes shown in volcano figure in SD-mutant ALKBH5 overexpressing cells with or without H_2_O_2_ treatment. Almost all the transcripts show negligible expression changes. (**I**) m^6^A peak visualization of key transcripts in DNA repair in control samples with or without H_2_O_2_ treatment. (**J**) qRT-PCR analyses showing that ROS markedly up-regulates transcription of the three selected target genes (FAAP20, ATRX and RRC2). (**K**) mRNA m^6^A methylation validation of the three selected target genes (FAAP20, ATRX and RRC2) by MeRIP analysis. (**L**) ALKBH5 RIP analyses showing that H_2_O_2_-induecd ROS dramatically decreases ALKBH5 enrichment at FAAP20, ATRX and RFC2 mRNAs. (**M–O**) mRNAs half-life of the three selected target genes (FAAP20, ATRX and RRC2), with or without ROS treatment.

To further determine how ROS induces stress response pathways through mRNA m^6^A methylation-mediated gene expression, we performed RNA sequencing (RNA-Seq) and m^6^A sequencing (m^6^A-Seq) analyses on untreated and H_2_O_2_-treated HEK293T cells expressing vector (vec samples) or ALKBH5 K86/K321R mutant. m^6^A-Seq revealed thousands of differential m^6^A peaks mediated by ROS (Figure 5A). As determined by the frequency distribution of differential m^6^A peaks across the length of mRNA transcripts, we found that ROS treatment specifically increased mRNA m^6^A in the 3′UTR regions in control samples (Figure 5A). However, overexpression of the ALKBH5 K86R/K321R mutant gene reduced m^6^A methylation in the 3′UTR region (Supplementary Figure S21A) and completely blocked ROS-induced mRNA m^6^A modification (Figure 5B). Consistent with m^6^A LC–QqQ–MS/MS quantitation experiments, ROS treatment in vec samples but not in ALKBH5 K86/321R mutant samples induced an obvious increase in m^6^A peaks (Figure 5C and D). We observed that the vast majority of increased m^6^A peaks were located in the 3′ UTR, and we also observed a moderate change of these hyper peaks in the 3′ UTR compared to other regions, while decreased m^6^A peaks were relatively evenly distributed along the mRNAs (Figure 5E and Supplementary Figure S21B). Notably, while ROS treatment in vec samples showed only a minor effect on ALKBH5 protein level, there was an apparent change in global m^6^A abundance in mRNA (Supplementary Figure S21C). We found that genes with significantly increased m^6^A modification of their mRNAs were enriched in multiple cellular processes including ribonucleoprotein complex biogenesis, translation, protein processing in the endoplasmic reticulum, DNA repair and DNA replication (Figure 5F). The analysis of differentially expressed genes in vec expressing cells showed that ROS treatment results in significant alteration in expression of 2157 genes, most of these up-regulated (Figure 5G), while in ALKBH5 K86/312R mutant samples, ROS treatment only leads to two genes with differential expression and none of them show differential peaks (Figure 5H). These results indicate that the ROS-induced global increase in mRNA m^6^A methylation and gene expression changes relies on ROS-induced ALKBH5 SUMOylation.

**Figure 6.**
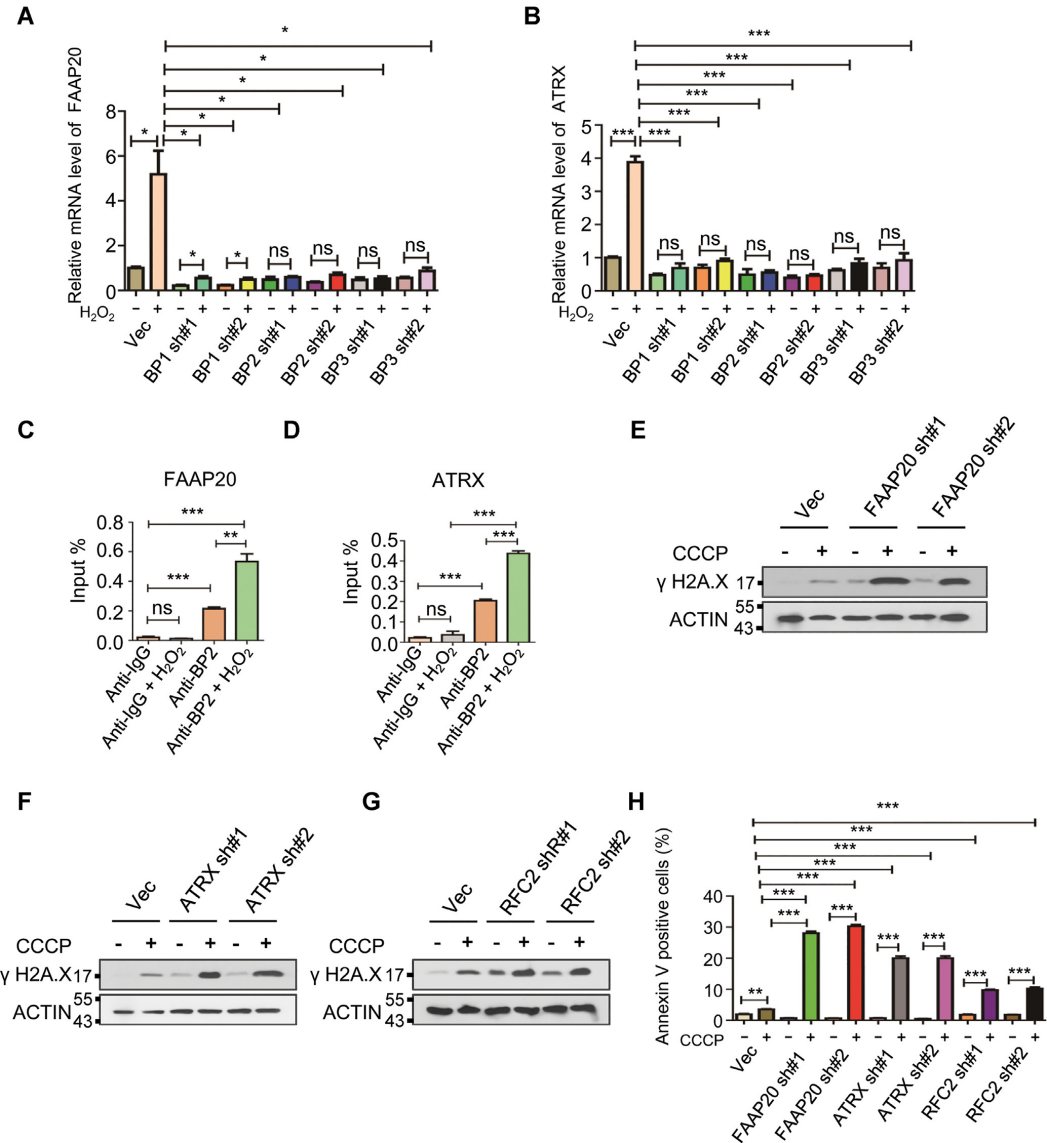
mRNA m^6^A modification enhances mRNA stability of FAAP20 and ATRX by IGF2BPs. (**A**, **B**) RT-PCR analysis showing that knockdown of IGF2BP1/2/3 completely blocks H_2_O_2_-induecd upregulation of FAAP20 and ATRX. (**C**, **D**) IGF2BP2 RIP analysis showing that H_2_O_2_ treatment significantly increases IGF2BP2 enrichment at mRNAs of FAAP20 and ATRX. **(E-G)** Western blot analyses indicating that knockdown of FAAP20, ATRX and RFC2 markedly increases CCCP-induced DNA damage in HEK293T cells. (**H**) Annexin V staining analysis showing that CCCP-induced cell apoptosis can be dramatically promoted by knockdown of FAAP20, ATRX and RFC2.

Combined with both global transcriptomic and epitranscriptomic (m^6^A methylomes) analysis, we identified 949 genes that have significantly changed levels of m^6^A methylation and transcription (Supplementary Figure S21D). Among the genes that are involved in DNA damage repair, ROS stress increased both transcript levels and m^6^A methylation at specific regions of the FAAP20, ATRX and RFC2 mRNAs in vector-expressing samples. By contrast, in ALKBH5 K86/321R mutant samples, ROS treatment led to negligible changes in transcript levels and m^6^A abundance (Figure 5I and Supplementary Figure S21E). Elevated transcription and mRNA m^6^A methylation of these three genes was further confirmed in control samples by RT-qPCR and methylated RNA immunoprecipitation (MeRIP) followed by RT-PCR analysis (Figure 5J and K, respectively). FAAP20, ATRX and RFC2 all play crucial roles in DNA damage repair (69–71). To further determine whether the altered mRNA m^6^A methylation and gene expression of these three genes is a consequence of ALKBH5-mediated demethylation, we performed a ALKBH5 RNA immunoprecipitation (RIP)-qPCR assay. As shown in Supplementary Figure S22A-S22C, ALKBH5 can be significantly enriched at mRNAs of FAAP20, ATRX and RFC2. More importantly, H_2_O_2_ treatment remarkably reduced ALKBH5 binding ability to FAAP20, ATRX and RFC2 mRNAs (Figure 5L). Furthermore, ROS-induced up-regulation of these genes related to DNA repair was only blocked by the SUMOylation-deficient mutant ALKBH5 but not wild-type ALKBH5 overexpression, suggesting that ALKBH5 SUMOylation plays an important role in ROS-induced expression of genes related to DNA damage repair (Supplementary Figure S23A–C). We next determined whether ROS affected mRNA stability of these three genes. By blocking new RNA synthesis with Actinomycin D, we measured the half-life of FAAP20, ATRX and RFC2 mRNAs as determined by qPCR analysis of transcripts of these three genes at different time points in the presence or in the absence of H_2_O_2_. The results showed that H_2_O_2_ treatment significantly promoted mRNA stability of FAAP20 and ATRX but not RFC2 mRNA (Figure 5M–O), suggesting that ROS-induced mRNA m^6^A methylation of FAAP20 and ATRX promotes FAAP20 and ATRX expression possibly by stabilizing their mRNAs. Taken together, these results suggest that ALKBH5 mediates ROS-induced expression of DNA repair genes partially by increasing their mRNA stability through reducing mRNA m^6^A demethylation of the respective RNAs.

Of interest, we also observed that transcription levels of both METTL3 and METTL14 can be induced by ROS in vec samples (Supplementary Figure S24A) but not in ALKBH5 knockout cells (Supplementary Figure S24B), or in cells with expression of ALKBH5 K86R/K321R mutant gene (data not shown). Western blot analysis also indicated that expression of ALKBH5 K86R/K321R mutant gene completely blocked ROS-induced protein levels of METTL3 and METTL14 (Supplementary Figure S24C). Thus, these data suggested that ROS promotes transcription of METTL3 and METTL14 mainly via ALKBH5 SUMOylation.

We found that deletion of ALKBH5 led to up-regulation of FAAP20, ATRX and RFC2 expression but not METTL3 and METTL14 (Supplementary Figure S24 D and S24F), indicating that ALKBH5-mediated METTL3 and METTL14 expression is dependent on ROS stress while ALKBH5 regulates FAAP20, ATRX and RFC2 in both homeostasis and stress conditions. In addition, we also observed that ALKBH5 knockout cells become more resistant to H_2_O_2_–induced DNA damage (Supplementary Figure S24E).

### m^6^A RNA modification enhances expression of FAAP20, ATRX by IGF2BPs-mediated mRNA stabilization

Despite numerous studies have shown that mRNA m^6^A modification promotes m^6^A marked mRNA turnover by YTHDF2-mediated mRNA degradation (16,72–75), several studies also demonstrated that mRNA m^6^A modification extends mRNA half-life by IGF2BPs-meiated mRNA stabilization (20,76). H_2_O_2_ treatment significantly stabilized FAAP20 and ATRX mRNAs but not RFC2 mRNA (Figure 5M-O), thus we further determined the underlying mechanism for m^6^A–mediated FAAP20 and ATRX mRNA stabilization. We knocked down IGF2BP1/2/3 in HEK293T cells by gene specific shRNAs (Supplementary Figure S25A-C). As shown in Figure 6A and B, IGF2BP1/2/3 knockdown completely blocked H_2_O_2_-induced up-regulation of FAAP20 and ATRX. In addition, IGF2BP2 RIP-qPCR analysis revealed that IGF2BP2 bound to FAAP20 and ATRX mRNAs and that H_2_O_2_ treatment significantly increased IGF2BP2 binding ability to FAAP20 and ATRX mRNAs (Figure 6C and D). To determine the significance of up-regulation of FAAP20, ATRX and RFC2 genes on DNA damage repair and cell survival in response to ROS, we knocked down all three genes individually by gene specific shRNAs in HEK293T cells (Supplementary Figure S26A–C). We showed that FAAP20, ATRX or RFC2 knockdown markedly increased CCCP-induced DNA damage and cell apoptosis (Figure 6E-H and Supplementary Figure S27). Collectively, these data suggest that the function of m^6^A methylation is mediated by IGF2BP1/2/3 in regulation of FAAP20 and ATRX mRNA stabilization and that FAAP20, ATRX and RFC2 are critical for DNA damage repair and cell survival in response to ROS.

### ROS stress response occurs in mouse bone marrow progenitor cells *in vivo*

**Figure 7.**
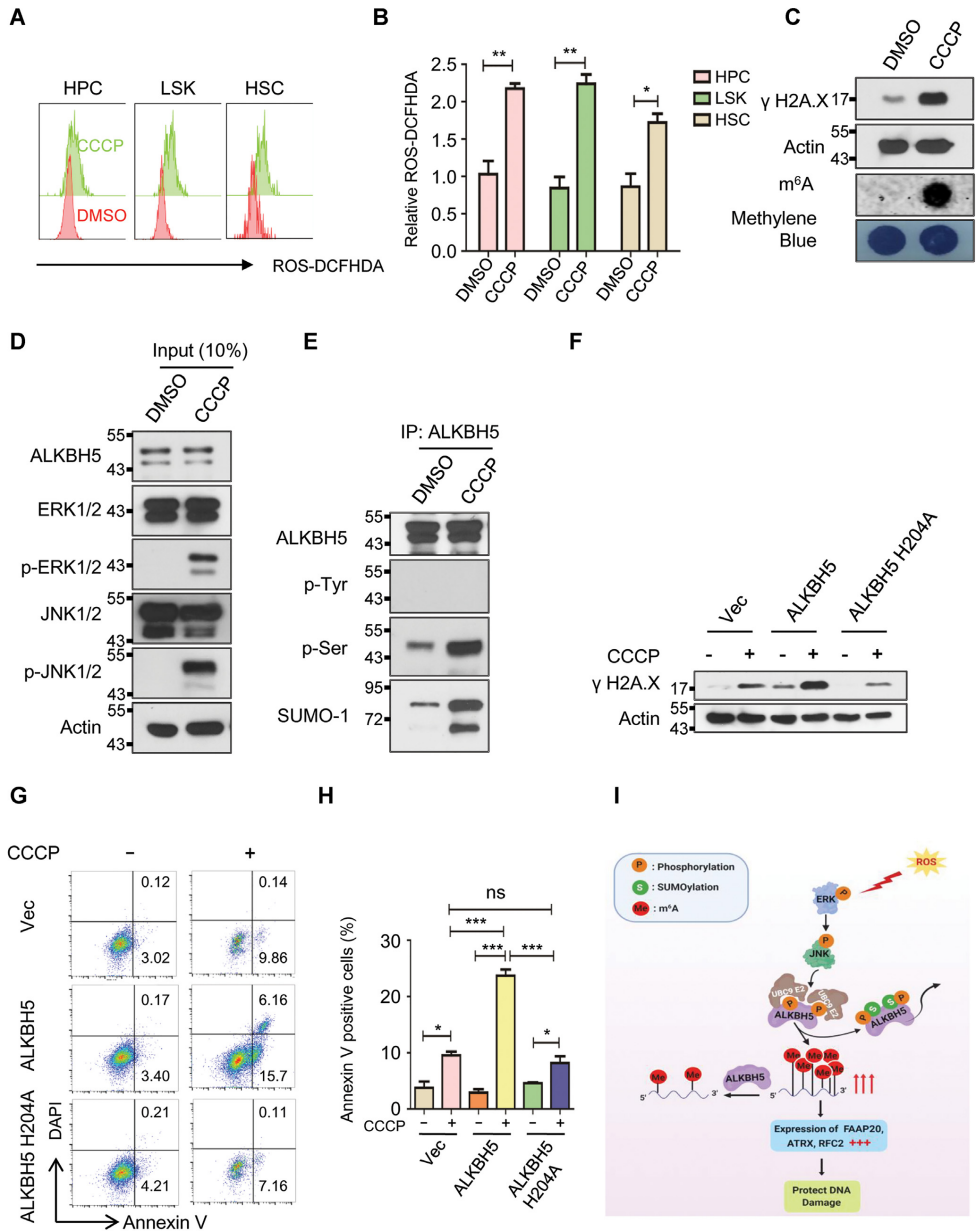
ROS stress activates the ERK/JNK/ALKBH5 PTMs/m^6^A methylation axis in mouse HSPCs *in vivo*. (A–E) CCCP or vehicle (DMSO) was intraperitoneally injected into three pairs of mice. And, all the mice were sacrificed 12 h after injection. (**A**, **B**) FACS analysis showing the effect of CCCP on endogenous ROS in mouse bone marrow stem/progenitor cells. (**C**) Western blot and dot-blot analysis showing phosphorylation of H2A.X and global mRNA m^6^A methylation levels in mouse bone marrow progenitor cells with or without CCCP treatment respectively. (**D**, **E**) Denaturing IP analysis indicates that ALKBH5 phosphorylation and SUMOylation can be dramatically induced by CCCP injection in mouse bone marrow progenitor cells. (**F**) Western blot analysis showing CCCP-induced DNA damage can be significantly enhanced by wild-type but not enzymatic mutant ALKBH5 overexpression in HPC-7 cells. (**G**) Annexin V staining analysis showing that ALKBH5 overexpression markedly facilitates CCCP-induced cell apoptosis in HPC-7 cells. (**H**) Histograms showing the summary and statistical analysis of G. (**I**) Working model of ROS mediated regulation of DNA repair genes. ROS stress activates ERK/JNK signaling resulting in phosphorylation of ALKBH5 at serine residues S87 and S321. ALKBH5 phosphorylation facilitates its SUMOylation at lysine residues K86 and K321, leading to the inhibition of its m^6^A demethylase activity and increase m^6^A modification in mRNAs of DNA damage repair related genes, such as FAAP20, ATRX and RFC2. As a consequence, increased expression of DNA repair genes protects cells from ROS- induced DNA damage.

Finally, we examined whether the activation of ERK/JNK/ALKBH5-PTMs/m^6^A axis occurs in primary hematopoietic stem/progenitor cells *in vivo* in response to ROS. As shown in Figure 7A, 7B and Supplementary Figure S28A and S28B, CCCP treatment markedly induces ROS in bone marrow hematopoietic stem cell enriched population (LSKs, Lin^–^c-kit^+^sca1^+^), hematopoietic progenitor cells (HPCs, Lin^–^c-kit^+^) as well as lineage negative progenitor cells (Lin^–^). Notably, CCCP-induced endogenous ROS significantly induced DNA damage in hematopoietic progenitor cells as evidenced by increased phosphorylation of H_2_AX as well as global mRNA m^6^A methylation (Figure 7C). Consistent with our observation in human cell lines, CCCP-induced ROS activated ERK/JNK phosphorylation as well as ALKBH5 phosphorylation and SUMOylation in bone marrow progenitor cells *in vivo* in mice (Figure 7D and E). More importantly, CCCP-induced DNA damage and cell apoptosis can be significantly facilitated by wild-type ALKBH5 but not enzymatic mutant ALKBH5 (ALKBH5 H204A) overexpression in mouse hematopoietic precursor cell-7 (HPC-7) cells, suggesting that ALKBH5-mediated ROS response depends on its m^6^A demethylase activity in mouse hematopoietic progenitor cells (Figure 7F–H). Taken together, these results suggest that ROS- induced ERK/JNK/ALKBH5 PTMs/m^6^A methylation axis plays an important role in the maintenance of genome integrity of hematopoietic stem/progenitor cell under physiological condition *in vivo* in response to ROS.

## DISCUSSION

Reactive oxygen species (ROS)-induced oxidative stress causes extensive cellular damage, and it is one of the major threats to cellular and organismal integrity (77). Here, we provide first evidence showing that mRNA m^6^A levels are markedly up-regulated in response to both H_2_O_2-_induced exogenous ROS and CCCP-induced endogenous ROS. We show that the up-regulation of mRNA m^6^A levels play an essential role in protecting cells from ROS-induced DNA damage and cell death. In addition, we uncovered a previously unrecognized mechanism that lead to up-regulation of mRNA m^6^A levels in response to ROS.

### Interplay between ERK/JNK signaling pathway and post-translational modifications of ALKBH5 mediate ROS-induced mRNA m^6^A methylation

Emerging data suggest a pivotal role of m^6^A methylation in response to environmental stressors (17,26,27). Hypoxic stress induces ALKBH5 expression (28) whereas m^6^A RNA methylation has been involved in guiding alternative translation of mRNA during the integrated stress response (29). Earlier study has shown that METTL3 was required for relocalization of DNA polymerase κ to DNA damage sites in response to ultraviolet light induced DNA damage (30). How signaling pathways mediate environmental or endogenous stress-induced alteration of mRNA m^6^A modification remains poorly understood. Numerous studies suggest that global SUMOylation is significantly induced by oxidative stress. In addition, a number of SUMO substrates have been identified in response to ROS stress (78,79). Oxidative stress also up-regulates global SUMOylation by cysteine thiol oxidation of the SENP1 and SENP2 catalytic domain, which leads to temporal inactivation of both enzymes (49). However, induction of SUMOylation of ALKBH5 in response to oxidative stress has not been reported.

We showed that ROS activates the extracellular regulated protein kinase1/2 (ERK1/2), subsequently leading to activation of c-Jun N-terminal kinase (JNK). As a consequence, activated JNK promoted serine but not tyrosine and threonine phosphorylation of ALKBH5. Phosphorylated ALKBH5 triggered ALKBH5 SUMOylation, leading to inhibition of its m^6^A demethylase activity and a global increase in mRNA m^6^A methylation. Mechanistically, ALKBH5 phosphorylation enhanced the interaction between ALKBH5 and the SUMO E2 conjugating enzyme UBC9 and inhibited the interaction between ALKBH5 and desumoylase SENP1, thereby promoting its SUMOylation in response to ROS stress. More importantly, the identified ALKBH5 SUMOylation and phosphorylation sites are conserved among species (Supplementary Figure S29). Thus, our study identifies ERK/JNK/ALKBH5-PTMs/m^6^A methylation as a previously unrecognized signaling axis, which plays a key role in the maintenance of genome integrity in response to ROS in human cells, and shows the novel cross-talk between ERK/JNK signaling pathway and ALKBH5 PTMs (Figure 7I).

It was shown that methyltransferase METTL3, adaptor protein METTL14, and demethylase FTO dynamically regulate m^6^A RNA in cells in response to UV light exposure (30). By contrast, we found that ALKBH5 but not FTO plays a key role in regulating m^6^A mRNA level in response to ROS. We showed that ALKBH5 inhibition increased m^6^A mRNA methylation of METTL3 and METTL14, leading to up-regulation of both genes in response to ROS. These results suggest a positive feedback mechanism through which ALKBH5 inhibition-mediated increase in m^6^A mRNA level is further augmented by ALKBH5-mediated up-regulation of METTL3 and METTL14 in response to ROS. Our studies thus demonstrate that m^6^A methylation is regulated through distinct molecular mechanisms in cells exposed to different environmental stressors.

### ALKBH5-mediated regulation of mRNA m^6^A methylation is essential for ROS-induced DNA damage response

Oxidative stress causes approximately 10^4^ DNA lesions per cell per day in an organism. Thus, rapid and accurate repair of oxidative damage is critical for the maintenance of genome integrity to prevent long-term implications of aging and cancer (80). Here, we have established a key role of ALKBH5 SUMOylation in coordinating ROS-induced cellular damage response through synchronizing expression of thousands of genes involved in a variety of biological processes through increasing global mRNA m^6^A methylation, as determined by m^6^A-seq and RNA-seq. Our results show that inhibition of mRNA m^6^A methylation enhances ROS-induced DNA damage and apoptosis. At a molecular level, DNA repair genes were among those significantly induced by ALKBH5 inhibition in response to ROS. They included FAAP20, ATRX, and RFC2 that are critical for DNA damage repair (69–71). ATRX plays a critical role in chromatin reconstitution, DNA repair synthesis and homologous recombination as well as non-homologous end joining (NHEJ) DNA repair (81,82) while FAAP20 is required for DNA inter-strand crosslink repair (83,84). RFC2 also plays a key role in both DNA replication and DNA repair (71). We further demonstrated that all three genes play an important role in repairing ROS-induced DNA damage. Mechanistic studies showed that ROS-induced mRNA m^6^A methylation of FAAP20 and ATRX promote their transcription levels by IGF2BP2-mediated mRNA stabilization. However, the underlying mechanism for m^6^A-mediated RFC2 mRNA up-regulation remains to be determined. In addition to up-regulation of genes associated with DNA damage repair, ROS-induced ALKBH5 inhibition also led to a significant up-regulation of METTL3 and METTL14 expression in HEK293T cells. However, depletion of ALKBH5 in the same cells did not increase the expression of METTL3 and METTL14, suggesting that ALKBH5 selectively targets METTL3 and METTL14 in response to ROS. It has been recently shown that METTL3-mediated DNA damage-associated RNA methylation promotes DNA damage repair by forming DNA-RNA hybrid and recruitment of DNA repair proteins to double-strand DNA breaks (85) Thus, ALKBH5 SUMOylation-mediated up-regulation of METTL3 expression may facilitate localization of METTL3 to ROS-induced DNA damage sites, thereby promoting DNA damage-associated RNA methylation, and DNA damage repair. Our study has identified an ALKBH5-SUMOylation-mediated mechanism that leads to up-regulation of METTL3 in response to stress. Importantly, we demonstrated the presence of ERK/JNK/ALKBH5-PTMs/m^6^A methylation signaling axis in HSPCs *in vivo* in response to endogenous ROS stress, suggesting a physiological role of this pathway on protecting HSPCs from ROS-induced DNA damage.

In conclusion, we have elucidated how mammalian cells coordinate signaling pathways, post-translational modification of ALKBH5 and m^6^A modification to react in a rapid and efficient manner to ROS-induced stress. Our study identified previously unrecognized molecular mechanisms by which mammalian cells enhance mRNA m^6^A methylation as a new layer of gene regulation to synchronize expression of thousands of genes involved in a variety of cellular processes to safeguard genomic integrity and to protect cells from stress-induced cell death (Figure 7I).

## DATA AVAILABILITY

The raw and processed m^6^A-Seq data have been deposited into NCBI Gene Expression Omnibus (GEO) database with accession number GSE144620 (https://www.ncbi.nlm.nih.gov/geo/query/acc.cgi?acc=GSE144620.

Links to a UCSC genome browser session displaying the uploaded sequence tracks are as follows:

http://genome.ucsc.edu/s/xiaolongC/ALKBH5_SUMOylation_FAAP20

http://genome.ucsc.edu/s/xiaolongC/ALKBH5_SUMOylation_ATRX

http://genome.ucsc.edu/s/xiaolongC/ALKBH5_SUMOylation_RFC2

The flow cytometry data has been deposited in FlowRepository (http://flowrepository.org/) with repository IDs FR-FCM-Z3HQ, FR-FCM-Z3HS, FR-FCM-Z3HT, and FR-FCM-Z3HU.

## Supplementary Material

gkab415_Supplemental_File
