# Supplementary material for: Post-translational modification of RNA m6A demethylase ALKBH5 regulates ROS-induced DNA damage response
Source: Nucleic Acids Res. 2021 May 28;49(10):5779–97. doi: 10.1093/nar/gkab415 (PMC8191756; doi:10.1093/nar/gkab415)
Supplement: gkab415_Supplemental_File [file gkab415_supplemental_file.pdf]

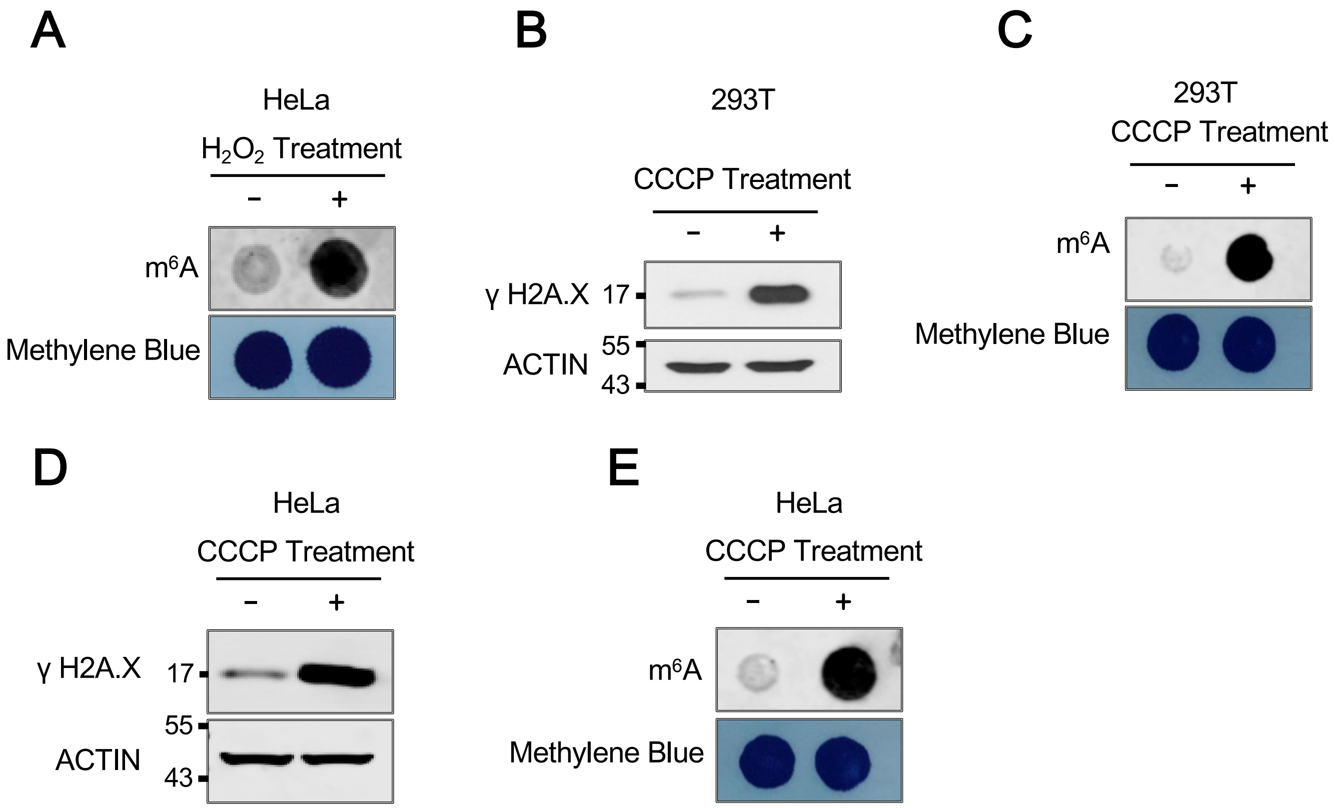

Supplementary Figure S1. ROS induces DNA damage and global increase in mRNA m^6A modification in HeLa and 293T cells. (A) Dot-blot analysis was performed to determine the effect of H_2O_2 treatment on global mRNA m^6A methylation in HeLa cells. (B) Western blot analysis showing protein levels of γ H2A.X in 293T cells with, or without CCCP treatment. (C) Dot-blot analysis showing the effect of CCCP treatment on global mRNA m^6A methylation in 293T cells. (D) Western blot analysis of γ H2A.X in HeLa cells. (E) Dot-blot analysis of global mRNA m^6A level in HeLa cells in the presence, or absence of CCCP.

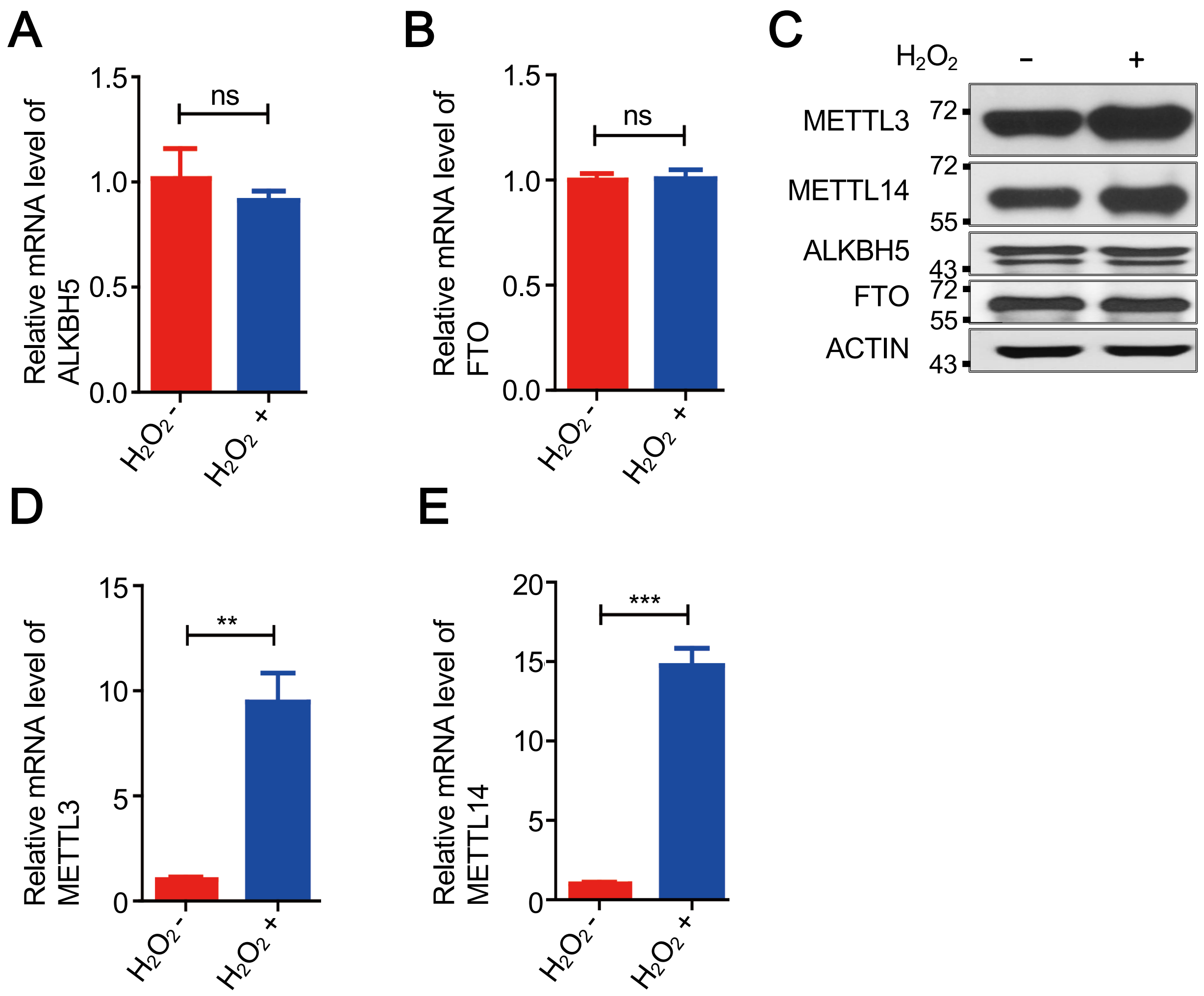

Supplementary Figure S2. ROS stress increases both transcription and protein level of mRNA m^6A writers. (A-E) HEK293T cells were treated with or without H_2O_2, RT-PCR (A, B, D, E) and western blot (C) analyses were performed to determine the effect of H_2O_2 -induced ROS on both transcription and protein level of mRNA m^6A writers and erasers.

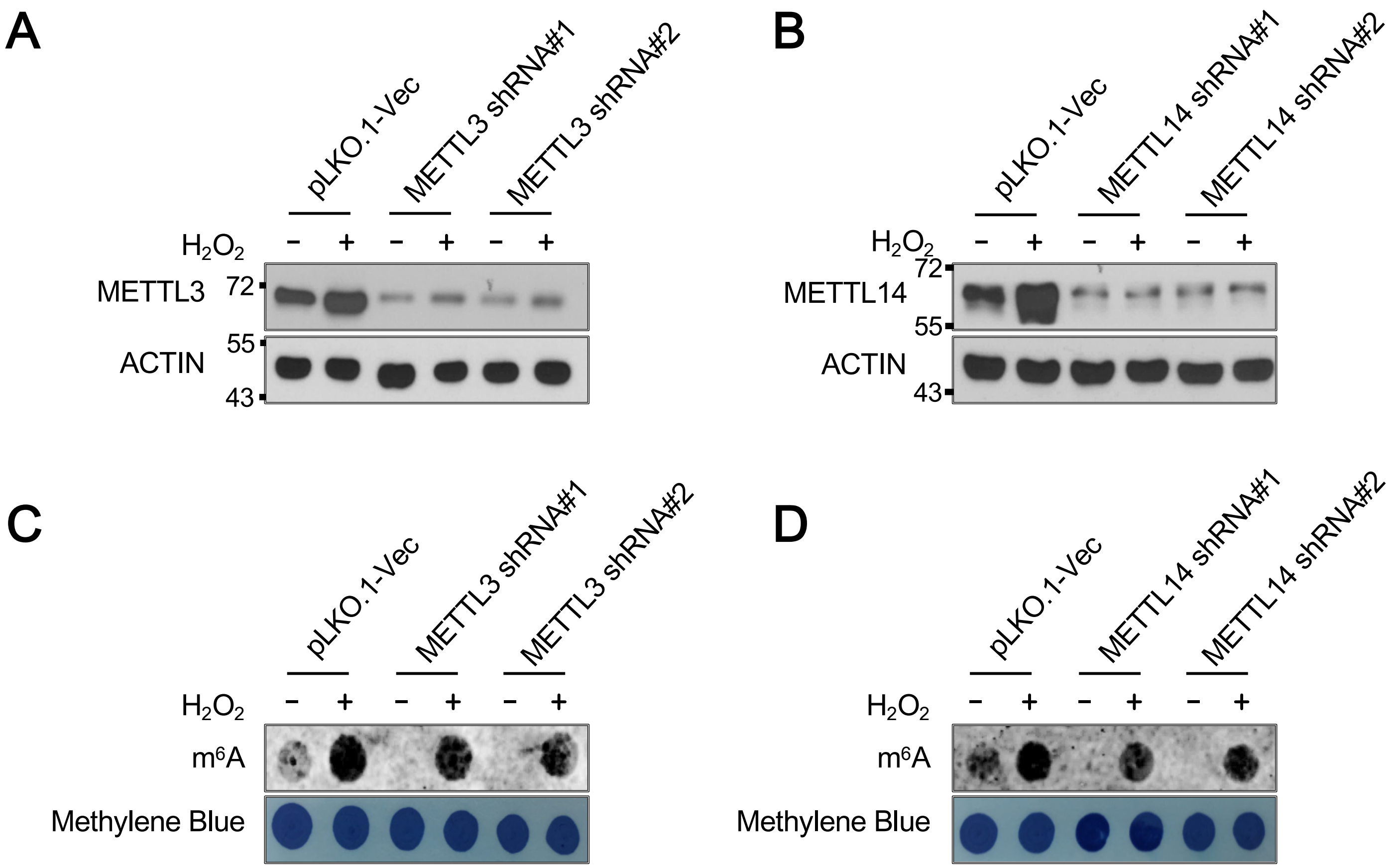

Supplementary Figure S3. Reducing the expression of METTL3 or METTL14 partially blocks ROS - induced global increase in mRNA m^6A methylation. (A-D) pLKO.1-Vec or shRNAs against METTL3 (A and C), or METTL14 (B and D) were transfected into HEK293T cells for 42 hours, then treated with or without H_2O_2. One half of the cells were used for the METTL3 (A) and METTL14 (B) knockdown efficiency detection, the rest of the cells for the dot-blot analyses (C and D).

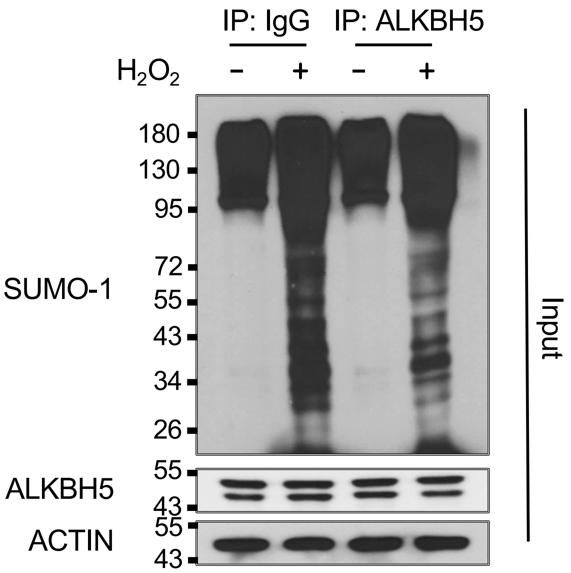

Supplementary Figure S4. H_2O_2 treatment significantly promotes ALKBH5 SUMOylation. Input of Figure 2A

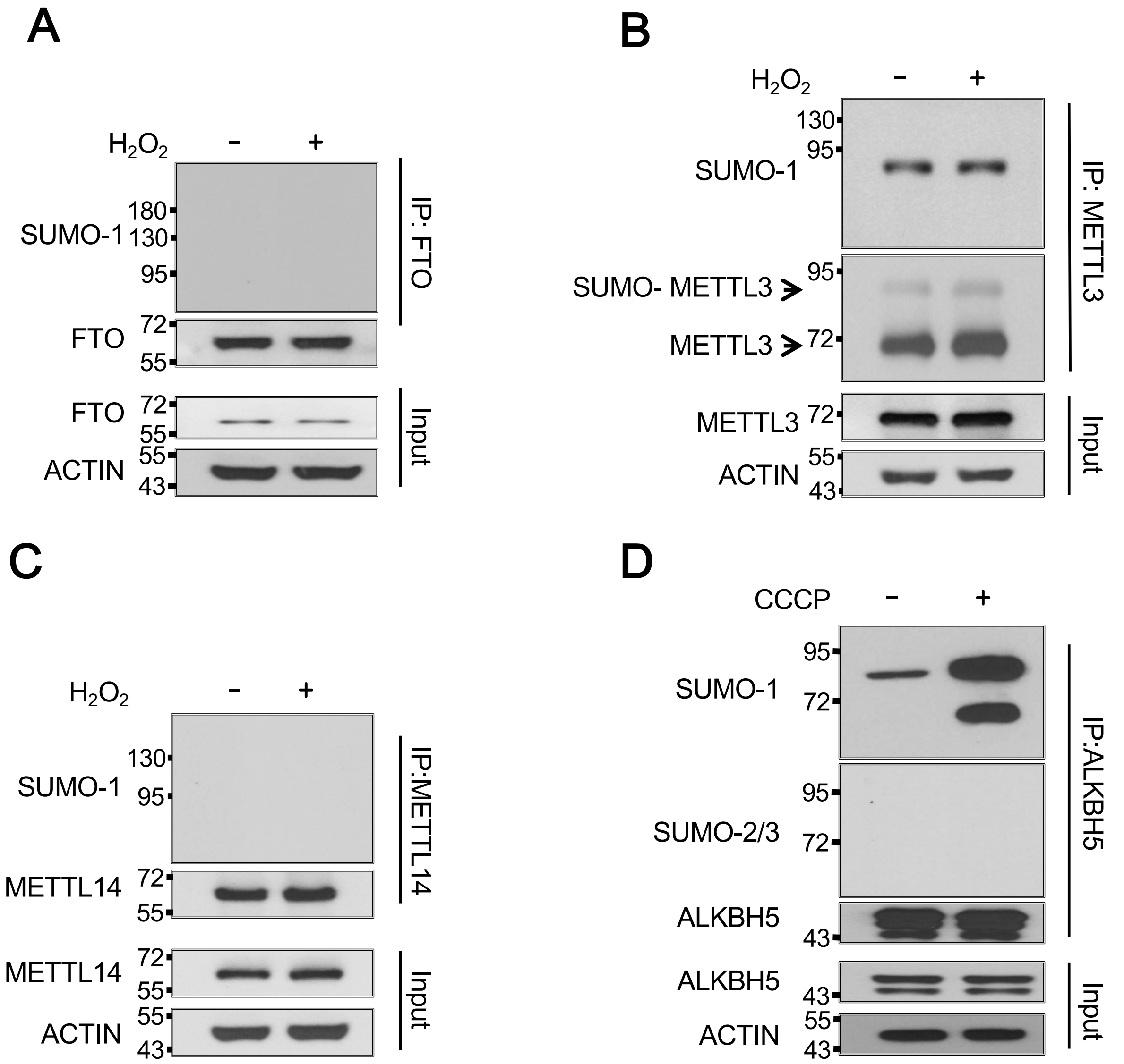

Supplementary Figure S5. ROS stress specifically promotes ALKBH5 SUMOylation. (A-C) SUMOylation of mRNA m^{6}A demethylase FTO, and m^{6}A writers METTL3 and METTL14 was determined by denaturing IP assay in HEK293T cells with or without H_2O_2 treatment, using antibodies as indicated. (D) denaturing IP assay showing that CCCP induces ALKBH5 SUMOylation in HEK293T cells.

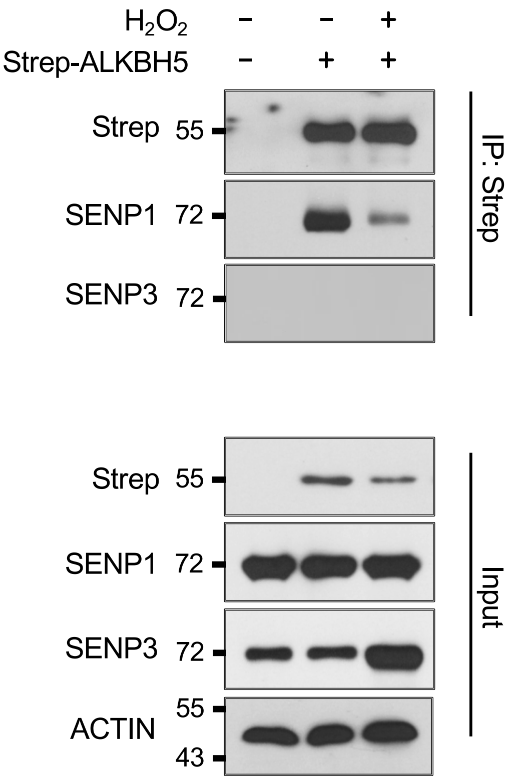

Supplementary Figure S6. H_2O_2 treatment markedly inhibits the interaction between ALKBH5 and SENP1. HEK293T cells stably expressing Strep-tagged ALKBH5 were treated with, or without H_2O_2 and Co-IP analysis was performed to determine the interaction between ALKBH5 and SENP1 and SENP3.

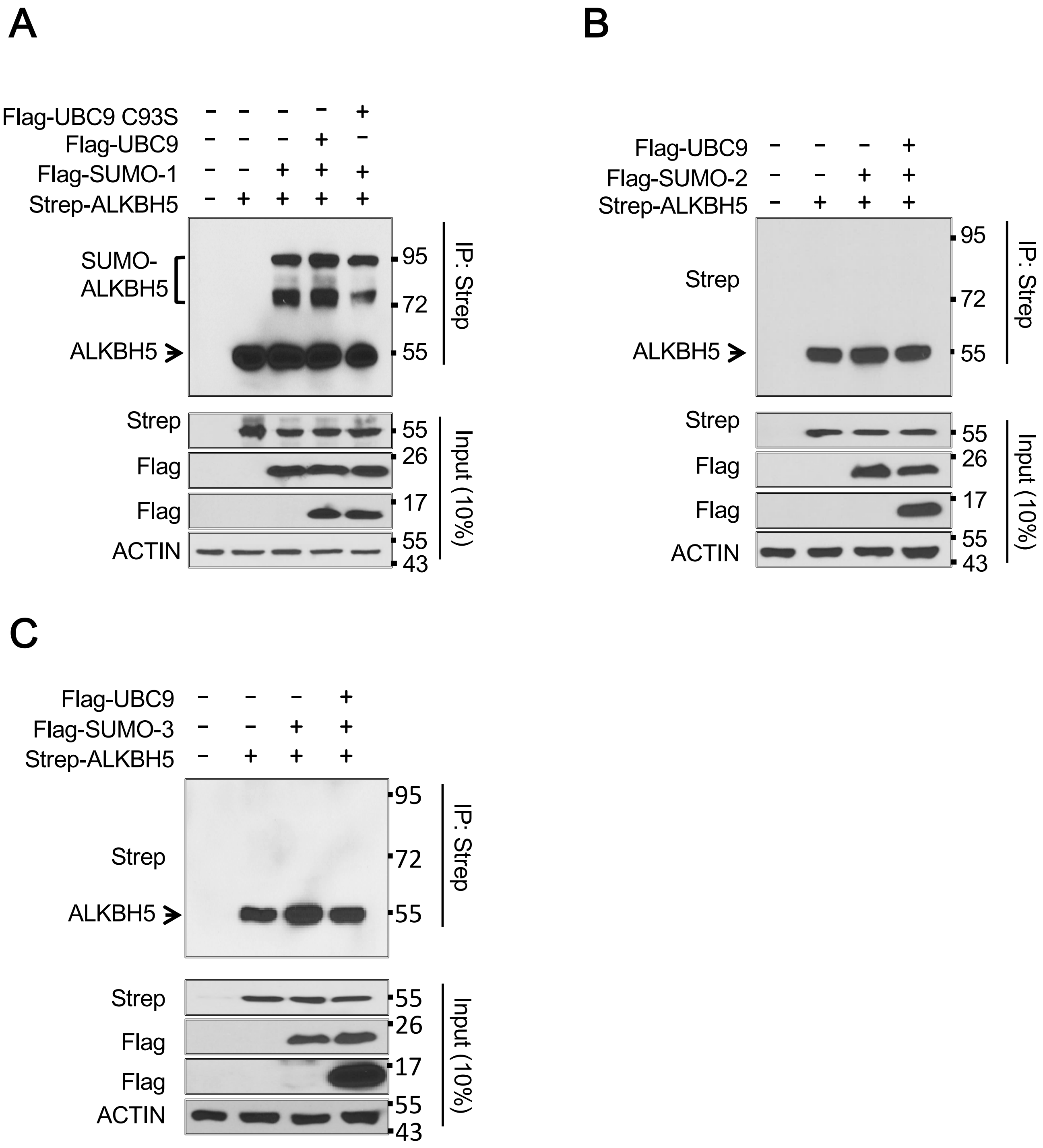

Supplementary Figure S7. ALKBH5 can be SUMOylated by SUMO-1 but not SUMO-2 and SUMO-3 in HEK293T cells. (A-C) HEK293T cells were solely transfected with Strep-ALKBH5, or co-expressed with wild-type Flag-UBC9 or SUMO E2-deficient mutant (Flag-UBC9 C93S) and Flag-SUMO-1 or Flag-SUMO-2 or Flag-SUMO-3, and ALKBH5 SUMOylation was revealed by denaturing IP assay.

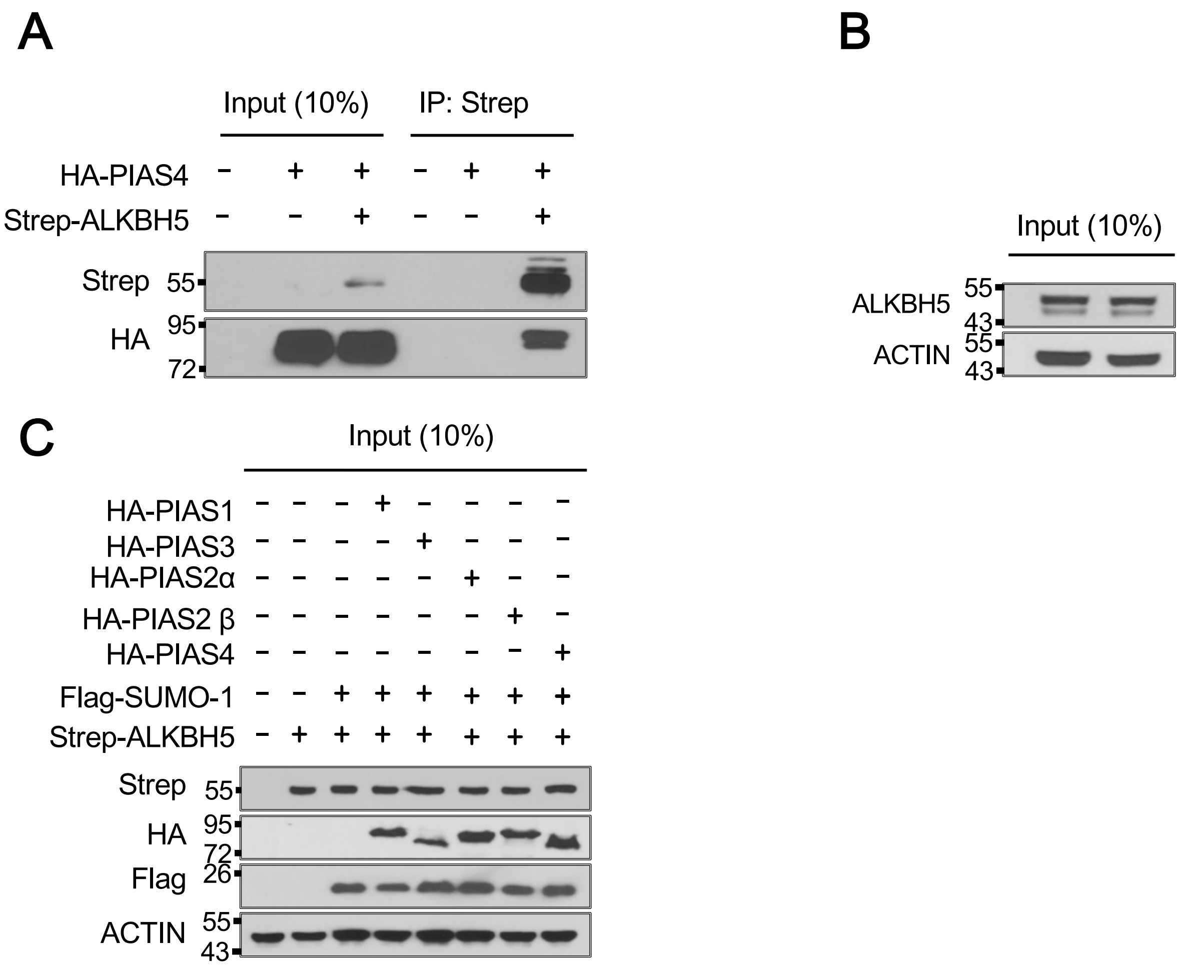

Supplementary Figure S8. SUMO E3 ligase PIAS4 interacts with ALKBH5. (A) HA-tagged PIAS4 was solely transfected or co-expressed with Strep-ALKBH5 into HEK293T cells. Two days after transfection, co-immunoprecipitation assay was performed to detect the interaction between PIAS4 and ALKBH5. (B) input of Figure 2D. (C) input of Figure 2E.

A

ALKBH5 TOP 5 Candidate Sumoylation sites

position	Peptide	Score
K390	GSPARKVKMRRH	37.23
K86	EEEARKVKSGIRQMR	34.039
K321	NNRDPALKPKRSHRK	31.801
K284	RAVIILRKTRLDAPR	23.676
K116	EVVSRAEKGLYNEHT	23.272

B

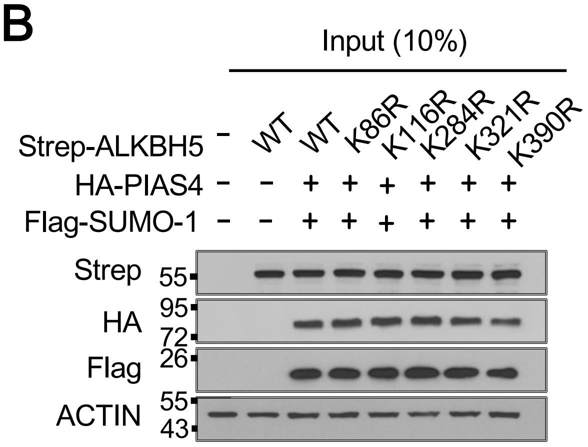

Supplementary Figure S9. ALKBH5 SUMOylation occurs at lysine residues K86 and K321. (A) The table showing the predicted ALKBH5 top 5 SUMOylation candidate sites by the GPS-SUMO and JASSA software, according to the SUMOylation consensus motif. (B) input of Figure 2F.

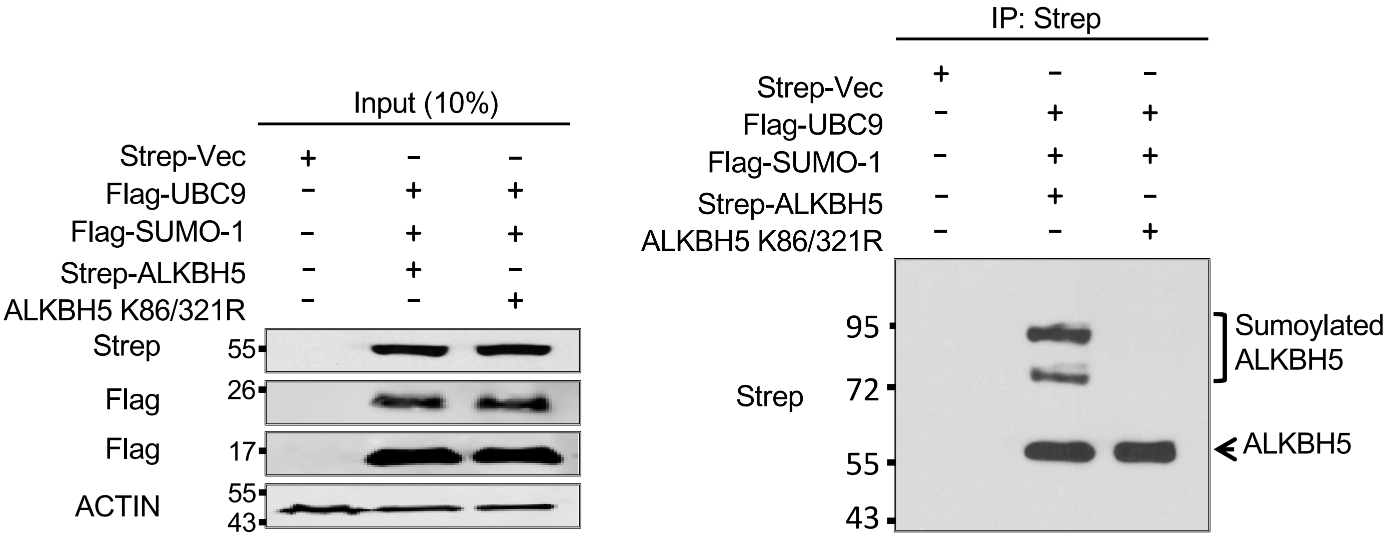

Supplementary Figure S10. Lysine residues K86 and K321 double mutation completely blocks ALKBH5 SUMOylation. HEK293T cells were transfected with Strep-ALKBH5 or Strep-ALKBH5 K86/K321 in the presence of co-expressed Flag-UBC9 and Flag-SUMO-1. Two days after transfection, denaturing IP assay was performed to determine SUMOylation of ALKBH5.

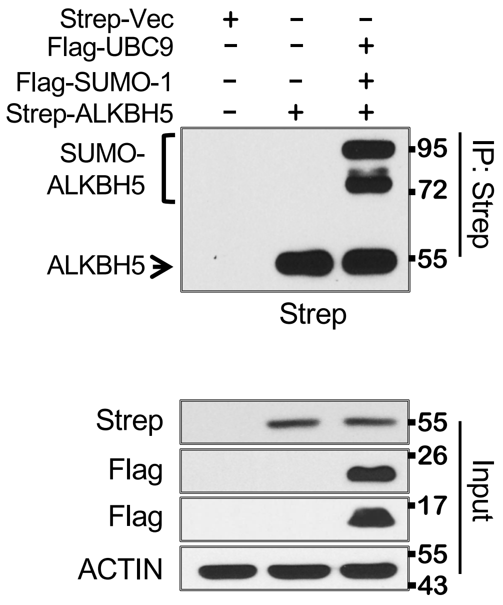

Supplementary Figure S11. Denaturing IP analysis suggesting UBC9 and SUMO-1 overexpression resulted in extensive SUMOylation of ALKBH5. Strep tagged ALKBH5 was transfected alone or co-expressed with Flag tagged UBC9 and SUMO-1 in HEK293T cells, 48 hours after transfection, the cells were collected and half of the cells were used for denaturing IP analysis and the rest of the cells were used for dot-blot analysis (Figure 2G).

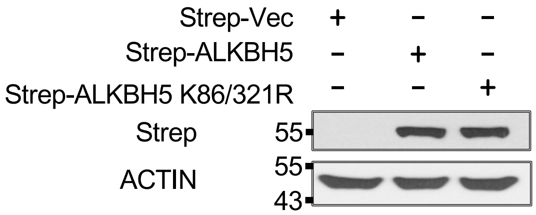

Supplementary Figure S12. Western blot analysis to confirm the expression of ectopically expressed plasmids. Strep tagged vector, wild-type ALKBH5 or SUMOylation-deficient mutant ALKBH5 (ALKBH5 K86/321R) were transfected into HEK293T cells, and 48 hours after transfection, half of the cells were used for western blot analysis to confirm the expression of the ectopically expressed plasmids. And the rest of the cells were used for dot-blot analysis (Figure 2H).

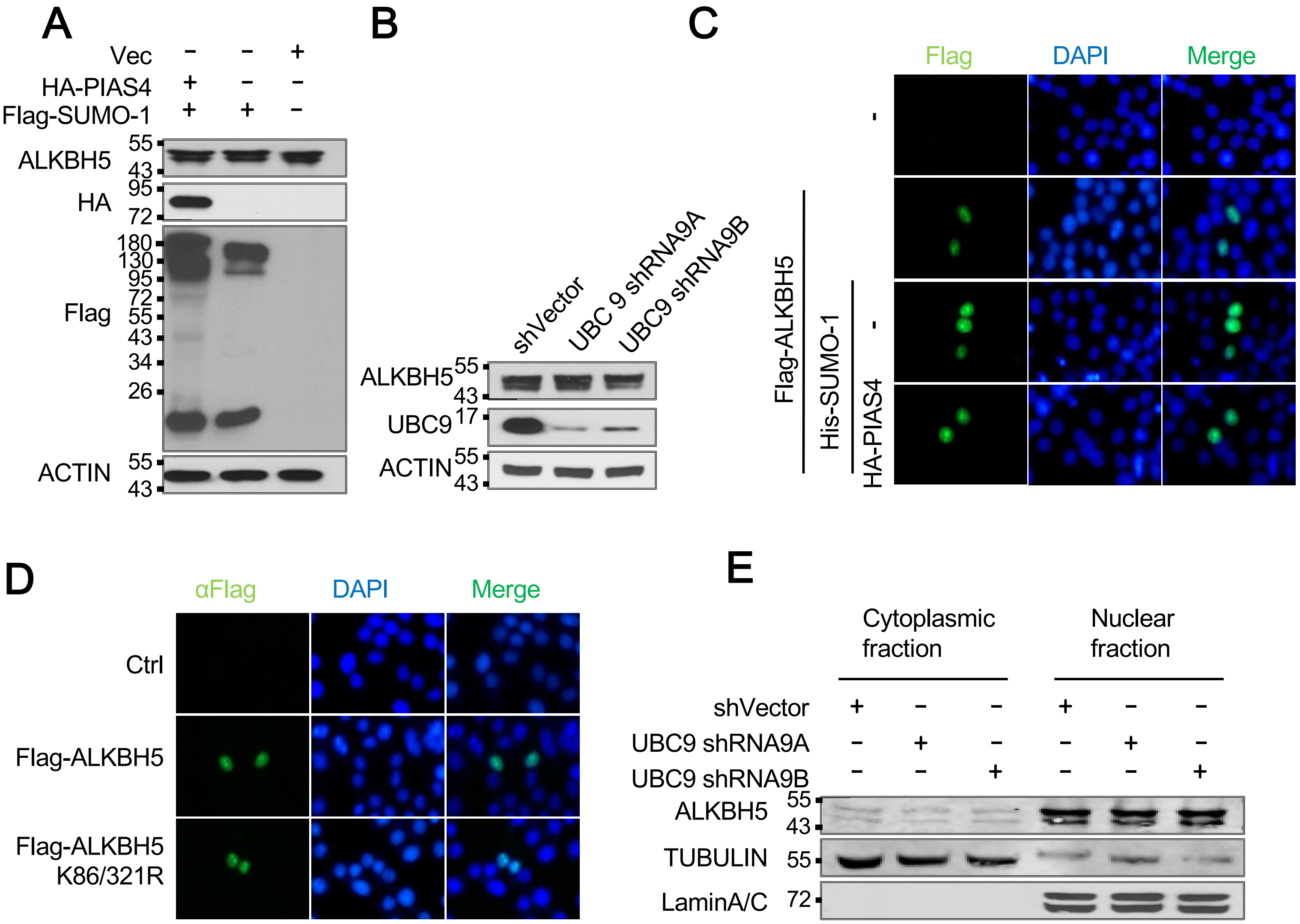

Supplementary Figure S13. ALKBH5 SUMOylation doesn't affect protein level and Sub-Cellular Localization. (A) Flag-SUMO-1 was solely transfected or co-expressed with HA-PIAS4 into HEK293T cells. 48 hours after transfection, cells were collected and western blot was carried out to determine the endogenous ALKBH5 protein level. (B) shvector or two shRNAs against UBC9 was transfected into HEK293T cells and 72 hours after transfection, western blot assay was performed, using antibodies as indicated. (C) The indicated plasmids were transfected into HeLa cells and 48 hours after transfection, immunostaining assay was performed to detect the ALKBH5 localization. (D) Flag-ALKBH5 or ALKBH5 SUMOylation deficient mutant Flag-ALKBH5 K86/K321 was transfected into HeLa cells and 48 hours after transfection the immunostaining analysis was performed by using anti-Flag antibody. (E) shvector or two shRNAs against UBC9 was transfected into HEK293T cells and 72 hours after transfection, the nuclear-cytoplasmic fraction assay was carried out to determine the effect of UBC9 knockdown on endogenous ALKBH5 sub-cellular localization.

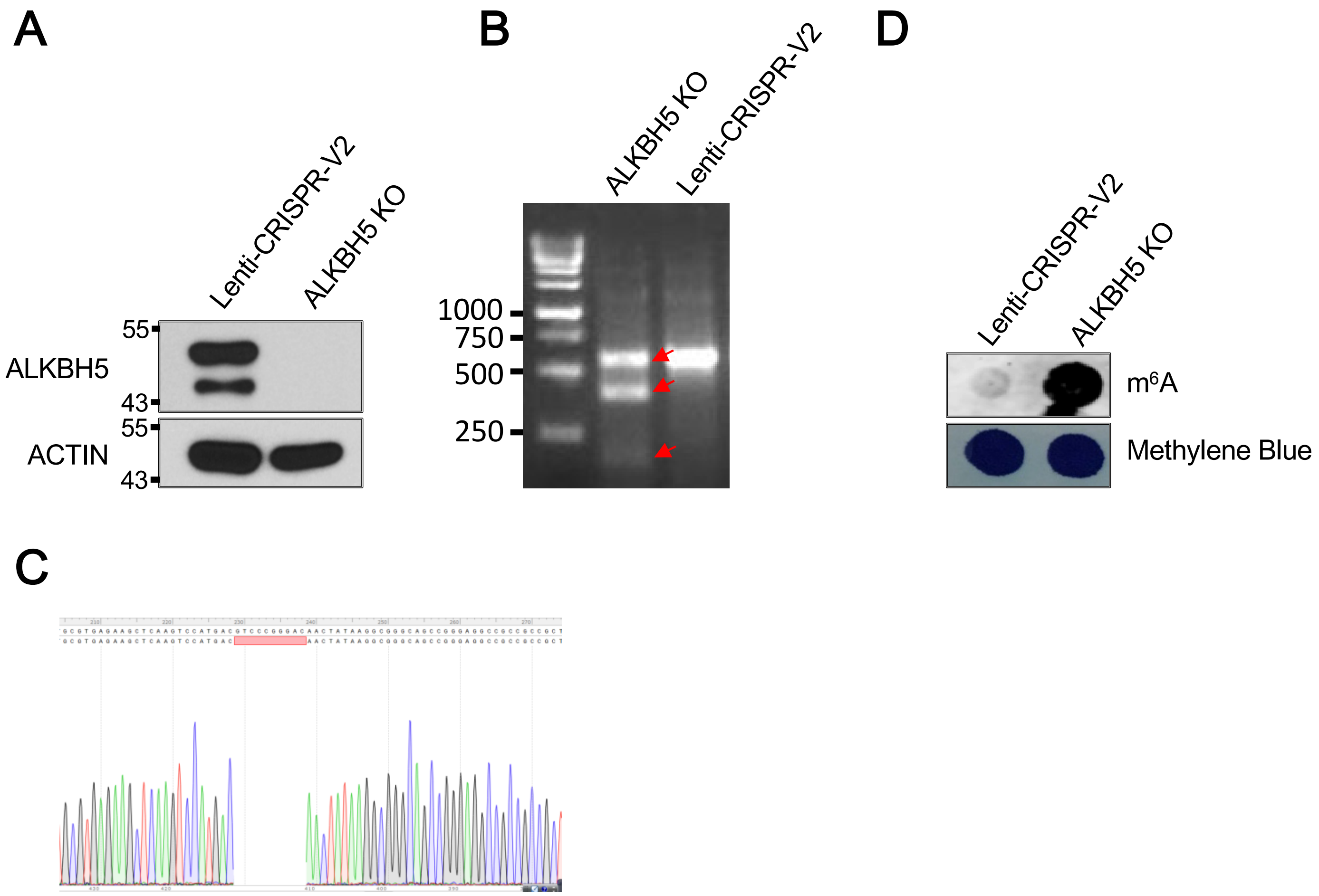

Supplementary Figure S14. Generation of ALKBH5 knockout cell line using CRISPR-Cas9 System. (A) Western blot analysis indicate that ALKBH5 is efficiently knocked out using CRISPR-Cas9 system in selected HEK293T monoclonal cell. (B) T7EI digestion assay showing that CRISPR-Cas9 - mediated genome editing works well in selected HEK293T monoclonal cell. (C) Endogenous ALKBH5 totally knockout was further demonstrated by DNA sequencing. (D) Dot blot analysis indicating that ALKBH5 knockout markedly increases mRNA m^6A modification.

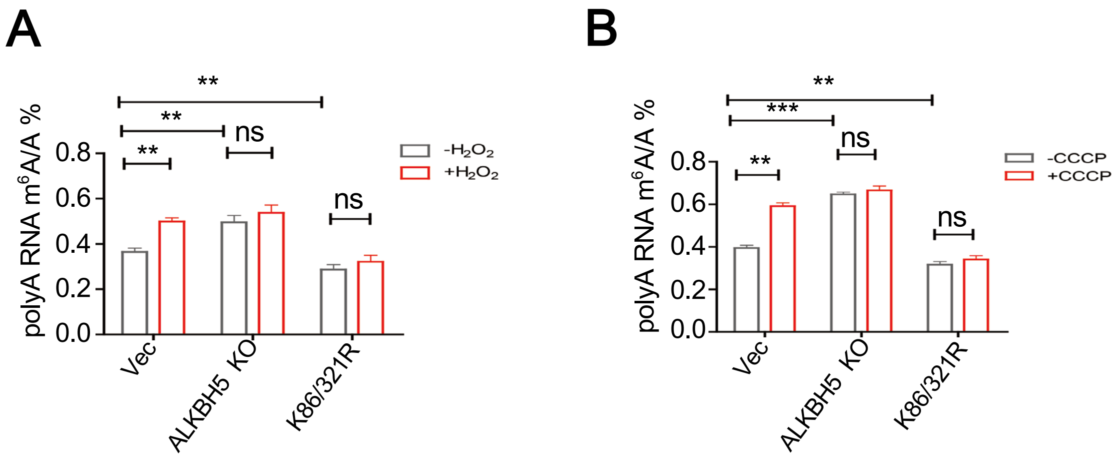

Supplementary Figure S15. (A and B) LC-MS/MS analyses indicating ROS induces global mRNA m^6A methylation via ALKBH5 SUMOylation. Control cells, ALKBH5 knockout cells, and SUMOylation-deficient mutant ALKBH5 overexpressing HEK293T cells were treated with or without H_2O_2 (A) or CCCP (B) for 6 hours, and subjected to LC-MS/MS analysis of mRNA m^6A methylation.

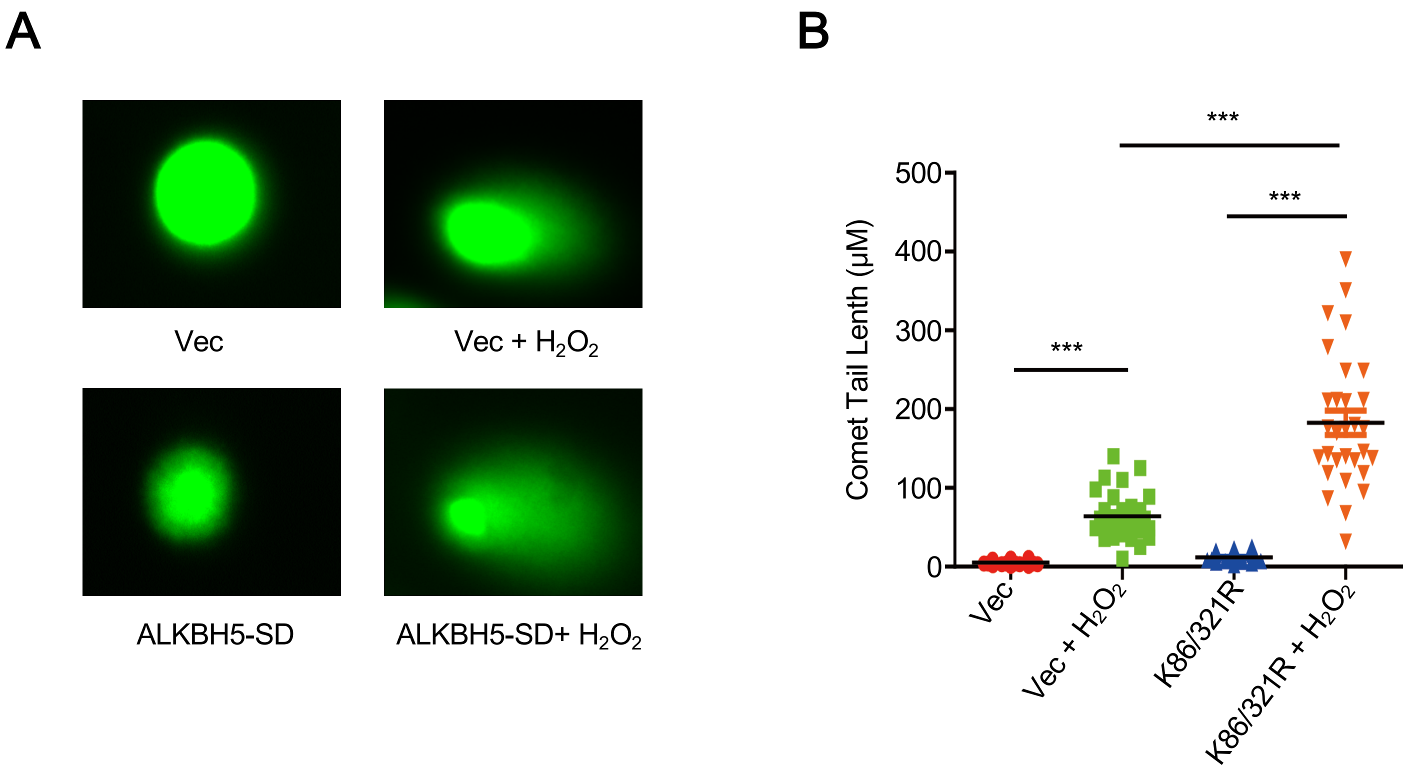

Supplementary Figure S16. Comet analysis showing that SUMOylation-deficient mutant ALKBH5 overexpression significantly promotes H_2O_2-induced DNA damage. (A) Comet analyses were performed in HeLa cells stably expressing vector or SUMOylation-deficient mutant ALKBH5 (K86/321R) with or without H_2O_2 treatment for six hours. (B) Quantification data of Figure S16A.

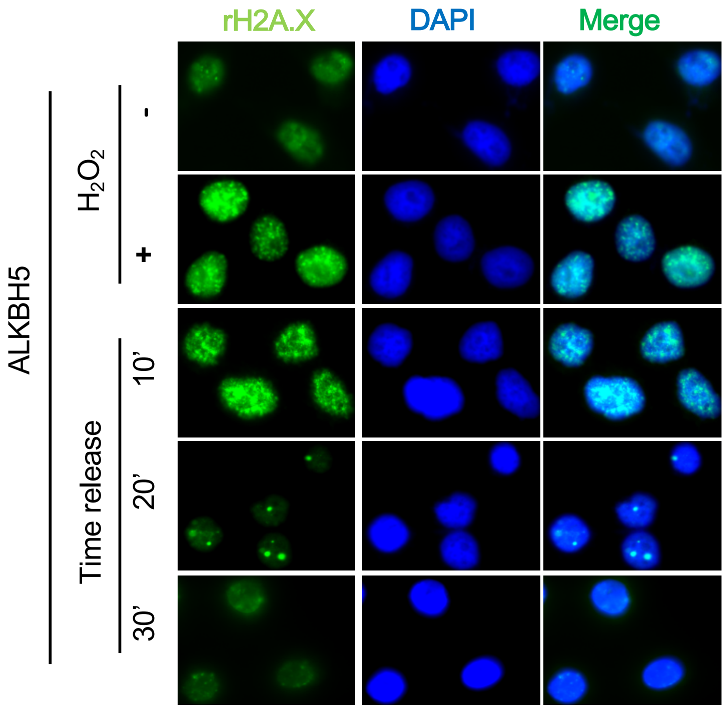

Supplementary Figure S17. H_2O_2 time release analysis showing that ALKBH5 overexpression delays H_2O_2–induced DNA damage repair. Immunostaining analysis suggesting that γ H2A.X foci still exists twenty minute after H_2O_2 release in HeLa cells stably expressing ALKBH5.

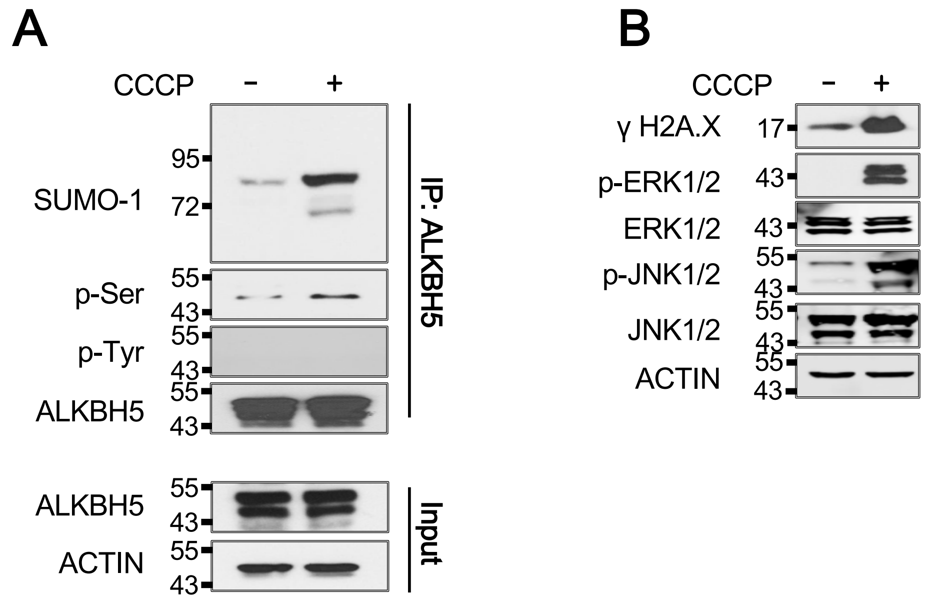

Supplementary Figure S18. CCCP-mediated internal ROS induces ALKBH5 phosphorylation and SUMOylation by activation of ERK/JNK signaling. (A) IP analysis was performed to determine the effect of CCCP treatment on ALKBH5 phosphorylation and SUMOylation in HEK293T cells treated with, or without CCCP, using antibodies as indicated. (B) Western blot analyses were performed to determine the effect of CCCP treatment on ERK/JNK signaling activation in HeLa cells.

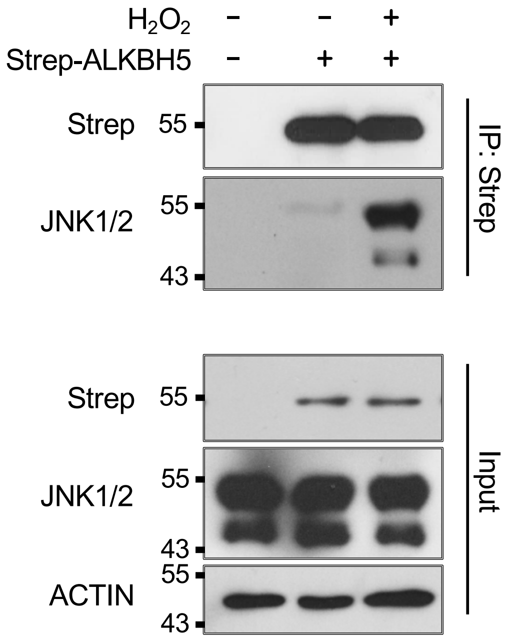

Supplementary Figure S19. H_2O_2 treatment significantly increases the interaction between JNK and ALKBH5. IP analysis was performed to determine the effect of H_2O_2 treatment on the interaction between ALKBH5 and JNK.

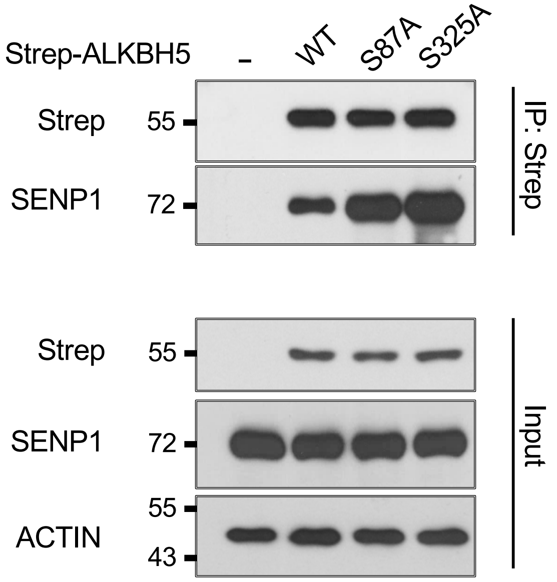

Supplementary Figure S20. ALKBH5 phosphorylation deficient mutation significantly enhances their interactions with SENP1. Co-IP analysis was performed to determine the effect of ALKBH5 phosphorylation sites mutation on the interaction between ALKBH5 and SENP1.

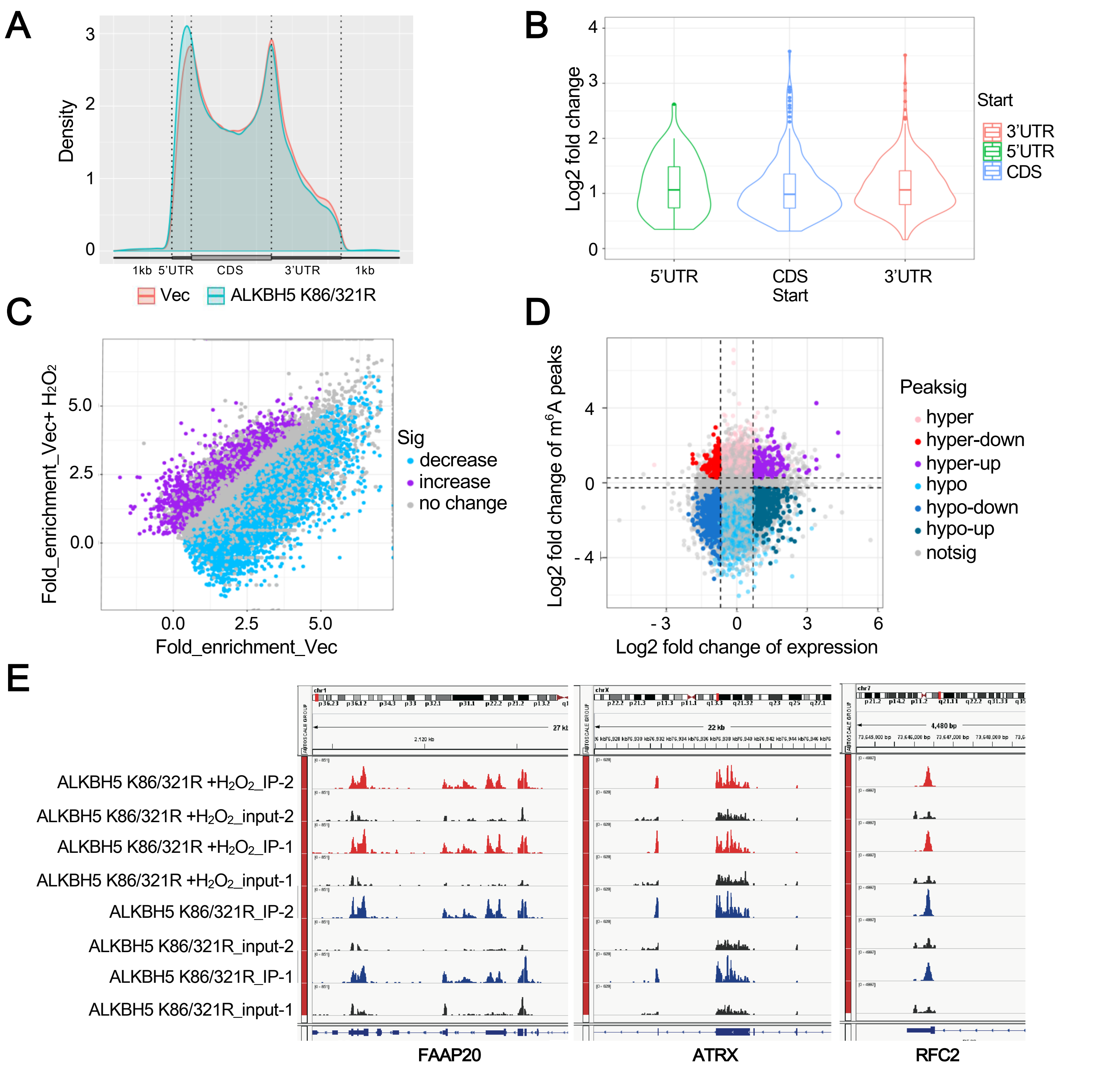

Supplementary Figure S21. mRNAs m^6A modification of DNA damage repair related downstream targets genes are not affected in SUMOylation-deficient mutant ALKBH5 overexpressed samples in the presence of ROS stress. (A) The frequency distribution of m^6A peaks across the length of mRNA transcripts shown by metagene in control samples and SUMOylation-deficient mutant ALKBH5 overexpression samples without H_2O_2 treatment. (B) Fold changes of hyper peaks from Figure 5C in different regions of mRNAs in control samples with or without H_2O_2 treatment. (C) Volcano figure for differential peaks in in control samples with or without H_2O_2 treatment. Including 5403 significantly decreased peaks (log2FC < 0, $p < 0.01$), 1998 significantly increased peaks (log2FC > 0, $p < 0.01$) and 13362 peaks without statistically significant changes. (D) Distribution of genes with a significant change in both m^6A methylation and overall expression in m^6A-seq assays in control samples with or without H_2O_2 treatment. Including 108 hyper-down peaks, 157 hyper-up peaks (both ATRX and FAAP20 fall into this category), 378 hypo-down peaks, and 304 hypo-up genes. (E) Peak visualizations of key transcripts in DNA repair in SUMOylation-deficient mutant ALKBH5 overexpression samples with or without H_2O_2 treatment.

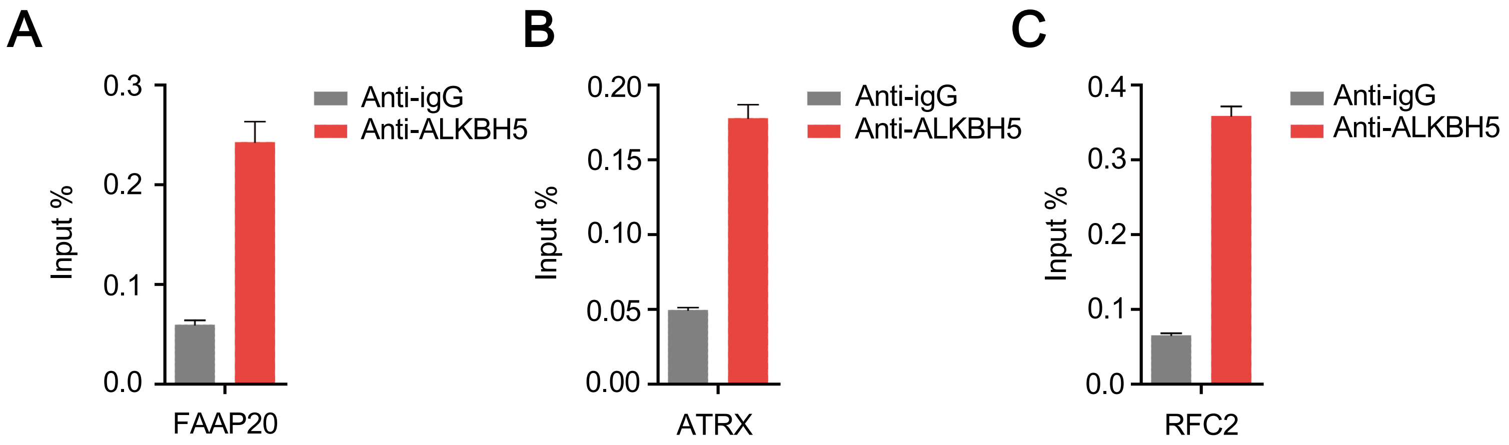

Supplementary Figure S22. ALKBH5 directly binds to mRNAs of FAAP20, ATRX and RFC2. (A-C) ALKBH5 RIP analyses were performed to detect ALKBH5 enrichment at mRNAs of the selected target genes, FAAP20, ATRX and RFC2.

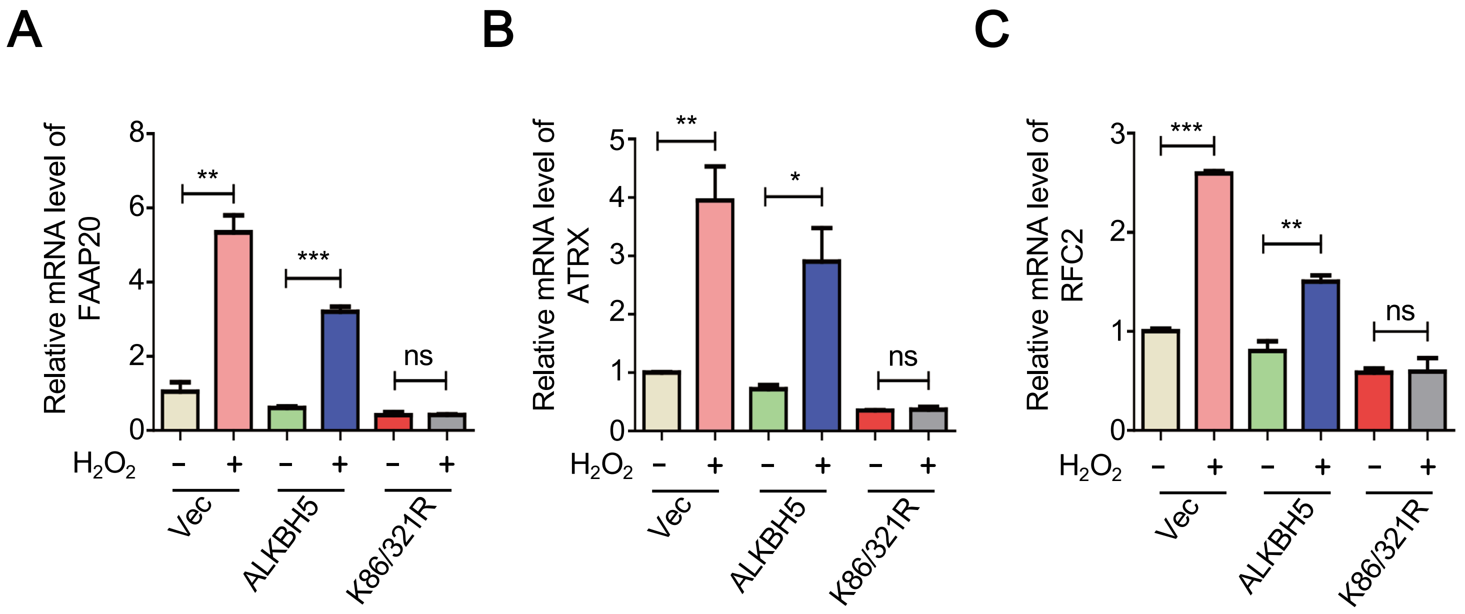

Supplementary Figure S23. SUMOylation of ALKBH5 plays a crucial role in ROS-induced up-regulation of DNA repair related genes.(A-C) RT-PCR analysis suggesting that SUMOylation-deficient mutant ALKBH5 overexpression completely blocks ROS-induced up-regulation of the target genes including FAAP20, ATRX and RFC2.

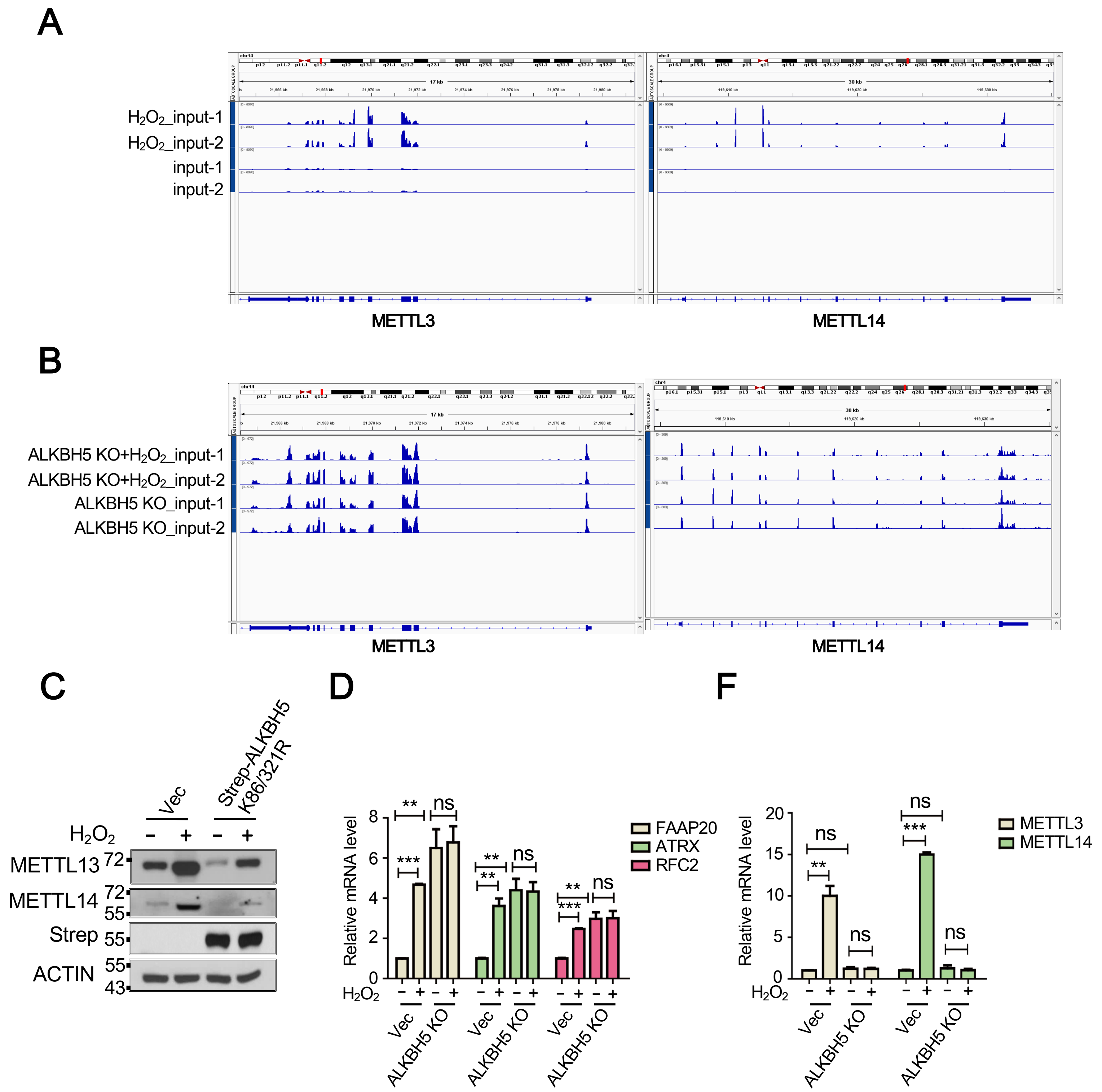

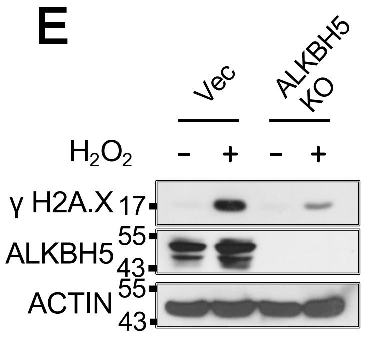

Supplementary Figure S24. ROS induces METLL3 and METTL14 up-regulation via ALKBH5 SUMOylation. (A and B) RNA-Seq reads peak visualizations of METTL3 and METTL14 in Vec (A), or ALKBH5 knockout samples (B) with or without H_2O_2 treatment. (C) Western blot analyses indicating H_2O_2 increases protein levels of METTL3 and METTL14 via ALKBH5 SUMOylation. (D) RT-qPCR analyses suggesting that ALKBH5-mediated upregulations of FAAP20, ATRX, RFC2 independent of H_2O_2. (E) Western blot analysis showing that ALKBH5 knockout inhibits H_2O_2–induced DNA damage in HEK293T cells. (F) RT-qPCR analyses indicating that ALKBH5-mediated upregulations of METTL3 and METTL14 depends on ROS stress.

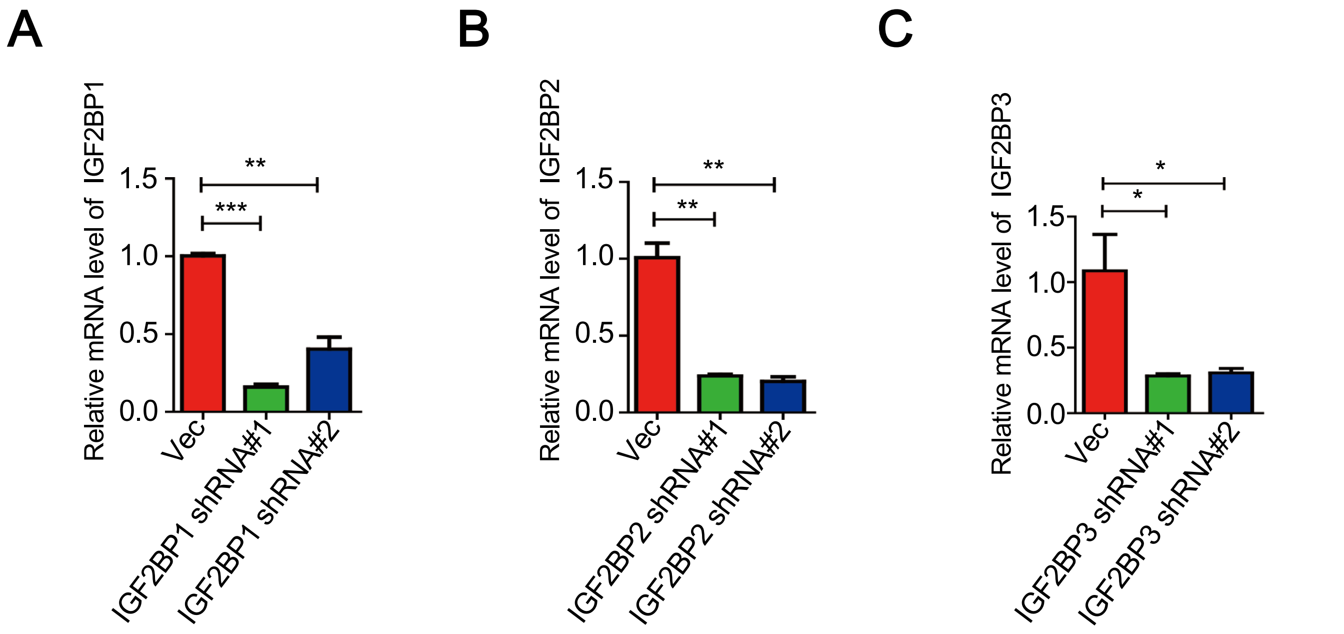

Supplementary Figure S25. RT-PCR analyses showing the knockdown efficiency of IGF2BP1/2/3. (A-C) RT-PCR analyses suggesting that IGF2BP1/2/3 can be efficiently knocked down by shRNAs.

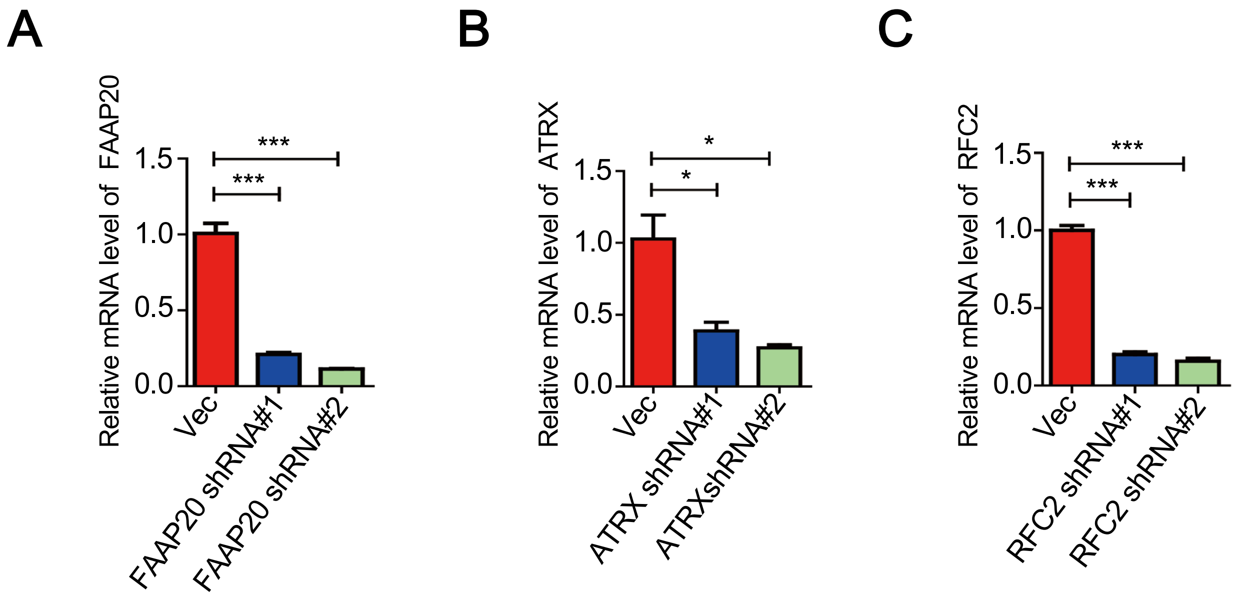

Supplementary Figure S26. RT-PCR analyses showing the knockdown efficiency of FAAP20, ATRX, and RFC2. (A-C) RT-PCR analyses suggesting that of FAAP20, ATRX, and RFC2 can be efficiently depleted by shRNAs-mediated knockdown.

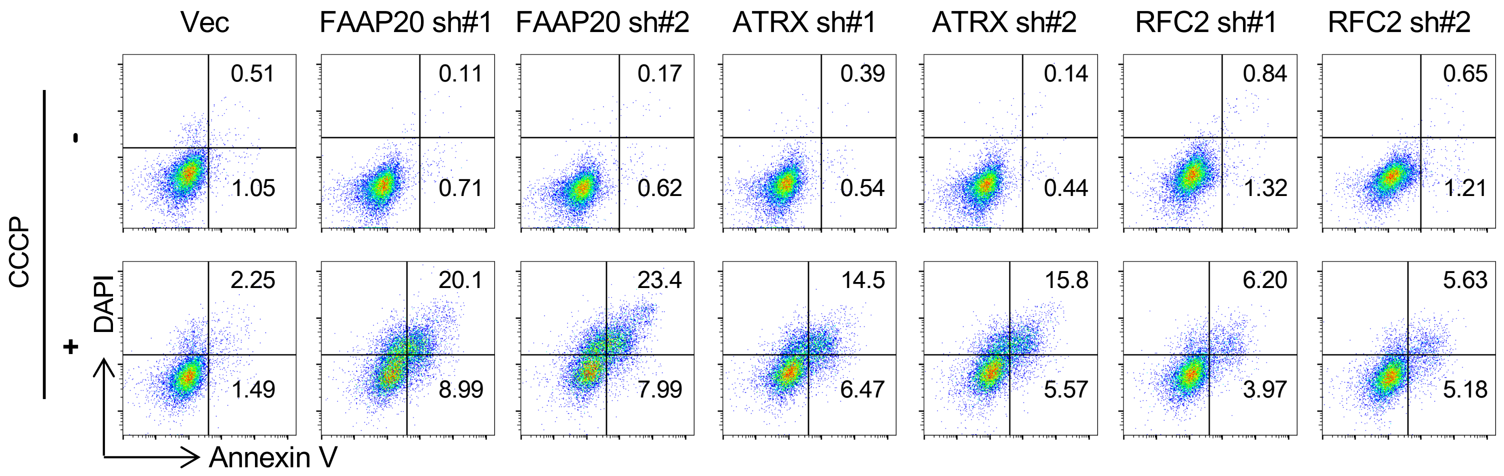

Supplementary Figure S27. Knockdown of FAAP20, ATRX, and RFC2 dramatically increases CCCP-induced cell apoptosis. Annexin V staining analyses were performed in HeLa cells stably expressing shvector, or shRNAs against FAAP20, ATRX, and RFC2 with or without CCCP treatment.

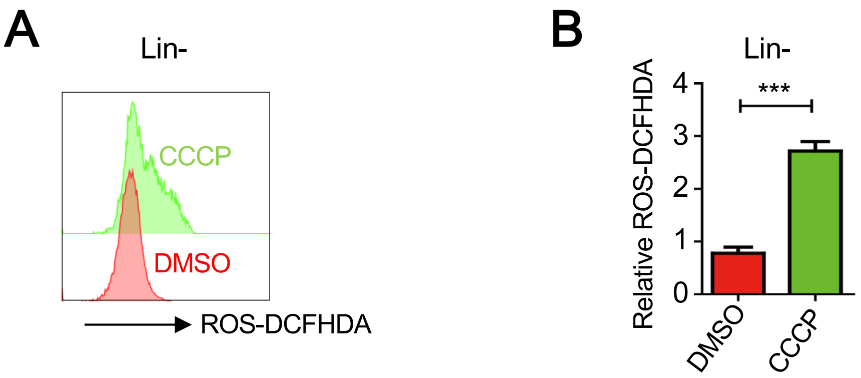

Supplementary Figure S28. CCCP injection induces endogenous ROS in lineage negative cells *in vivo*. (A) FACS analysis showing the effect of CCCP injection on endogenous ROS in mouse bone marrow lineage negative cells. (B) Quantification data of Figure S22A.

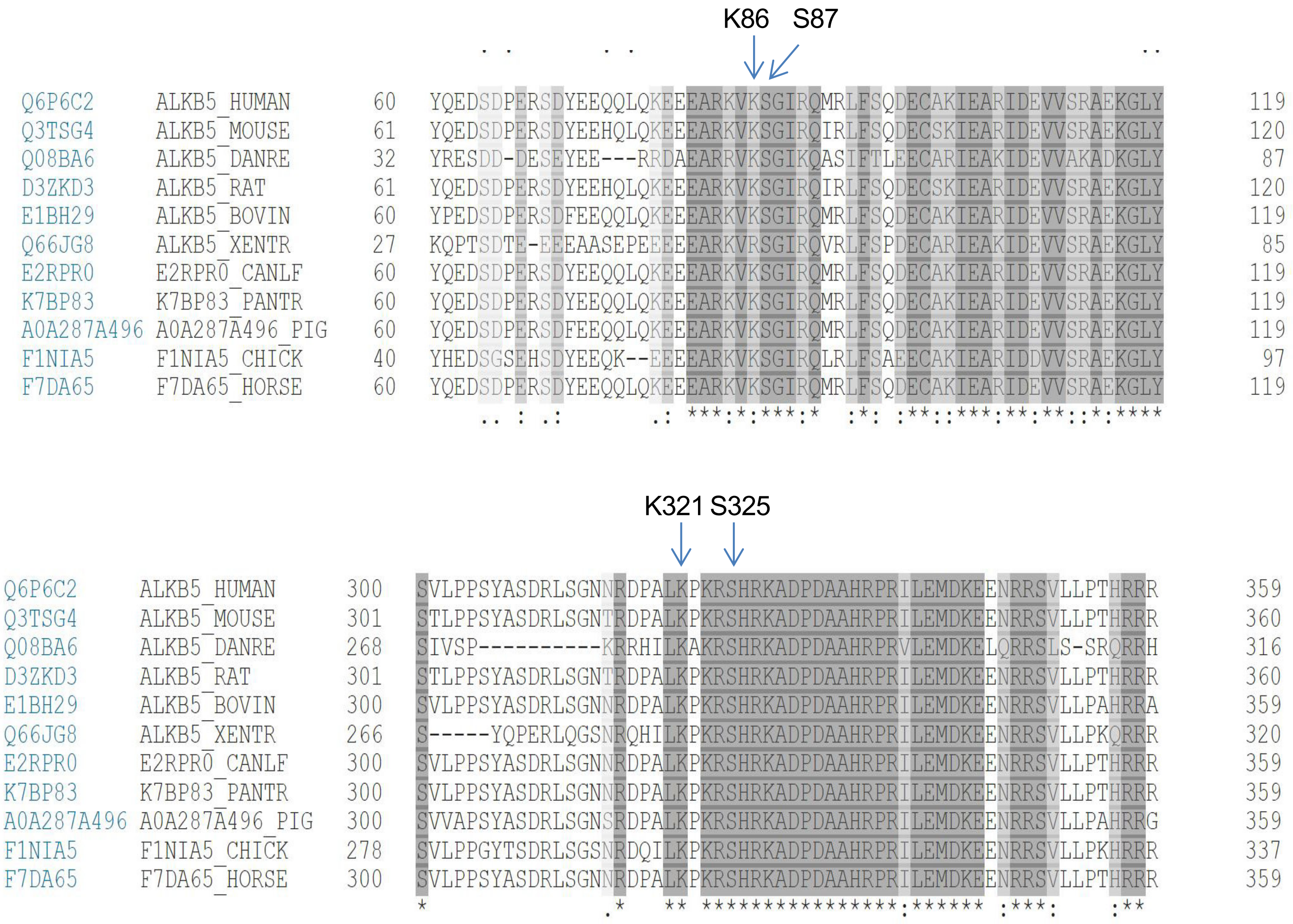

Supplementary Figure S29. ALKBH5 SUMOylation and phosphorylation sites are conserved among species. Uniprot website was used to perform ALKBH5 SUMOylation and phosphorylation sites alignments across different species.

Supplementary Figure Table S1. Sequences for RT-qPCR primers, gRNAs and shRNAs

RT-qPCR primers
hActin-5': CATGTACGTTGCTATCCAGGC
hActin-3': CTCCTTAATGTCACGCACGAT
hMETTL3-5': TTGTCTCCAACCTTCCGTAGT
hMETTL3-3': CCAGATCAGAGAGGTGGTGTAG
hMETTL14-5': AGTGCCGACAGCATTGGTG
hMETTL14-3':
GGAGCAGAGGTATCATAGGAAGC
hALKBH5-5': CGGCGAAGGCTACACTTACG
hALKBH5-3': CCACCAGCTTTTGGATCACCA
hFTO-5': ACTTGGCTCCCTTATCTGACC
hFTO-3': TGTGCAGTGTGAGAAAGGCTT
hFAAP20-5': AGGAGGGCACCTGGAATCC
hFAAP20-3': TCTTGCACATCCATGAAAGACAC
hATRX-5': GCTGAGCCCATGAGTGAAAG
hATRX-3': CGTGACGATCCTGAAGACTTG
hRFC2-5': GTGAGCAGGCTAGAGGTCTTT
hRFC2-3': TGAGTTCCAACATGGCATCTTTG
hIGF2BP1-5': GCGGCCAGTTCTTGGTCAA
hIGF2BP1-3': TTGGGCACCGAATGTTCAATC
hIGF2BP2-5':
AGTGGAATTGCATGGGAAAATCA
hIGF2BP2-3': CAACGGCGGTTTCTGTGTC
hIGF2BP3-5': TATATCGGAAACCTCAGCGAGA
hIGF2BP3-3': GGACCGAGTGCTCAACTTCT

gRNAs sequences
ALKBH5 gRNA-5': CACCGAGCTCAAGTCCATGACGTCC
ALKBH5 gRNA-3': AAACGGACGTCATGGACTTGAGCTC

Supplementary Figure Table S1. Sequences for RT-qPCR primers, gRNAs and shRNAs

shRNAs Sequences

hMETTL3 shRNA#1-5':
CCGGGCAAGTATGTTCACTATGAAACTCGAGTTTCATAGTGAACATACTTGCTTTTTG
hMETTL3 shRNA#1-3':
AATTCAAAAAGCAAGTATGTTCACTATGAAACTCGAGTTTCATAGTGAACATACTTGC
hMETTL3 shRNA#2-5':
CCGGGCTGCACTTCAGACGAATTATCTCGAGATAATTCGTCTGAAGTGCAGCTTTTTG
hMETTL3 shRNA#2-3':
AATTCAAAAAGCTGCACTTCAGACGAATTATCTCGAGATAATTCGTCTGAAGTGCAGC
hMETTL14 shRNA#1-5':
CCGGCCATGTACTTACAAGCCGATACTCGAGTATCGGCTTGTAAGTACATGGTTTTT
hMETTL14 shRNA#1-3':
AATTAAAAACCATGTACTTACAAGCCGATACTCGAGTATCGGCTTGTAAGTACATGG
hMETTL14 shRNA#2-5':
CCGGGCCGTGGACGAGAAAGAAATACTCGAGTATTTCTTTCTCGTCCACGGCTTTTT
hMETTL14 shRNA#2-3':
AATTAAAAAGCCGTGGACGAGAAAGAAATACTCGAGTATTTCTTTCTCGTCCACGGC
shUBC9A-5':CCGGAGCAGAGGCCTACACGATTTACTCGAGTAAATCGTGTAGGCCTCTGCTTTTTTG
shUBC9A-3': AATTCAAAAAAGCAGAGGCCTACACGATTTACTCGAGTAAATCGTGTAGGCCTCTGCT
shUBC9B-5': CCGGAGAAGTTTGCGCCCTCATAAGCTCGAGCTTATGAGGGCGCAAACTTCTTTTTTG
shUBC9B-3': AATTCAAAAAAGAAGTTTGCGCCCTCATAAGCTCGAGCTTATGAGGGCGCAAACTTCT
hIGF2BP1 shRNA#1-5':
CCGGGCAGTGGTGAATGTCACCTATCTCGAGATAGGTGACATTCACCACTGCTTTTTG
hIGF2BP1 shRNA#1-3':
AATTCAAAAAGCAGTGGTGAATGTCACCTATCTCGAGATAGGTGACATTCACCACTGC
hIGF2BP1 shRNA#2-5':
CCGGGCGGACTTGGAGAAAGTGTTTCTCGAGAAACACTTTCTCCAAGTCCGCTTTTTG
hIGF2BP1 shRNA#2-3':
AATTCAAAAAGCGGACTTGGAGAAAGTGTTTCTCGAGAAACACTTTCTCCAAGTCCGC
hIGF2BP2 shRNA#1-5':
CCGGAGTGAAGCTGGAAGCGCATATCTCGAGATATGCGCTTCCAGCTTCACTTTTTG
hIGF2BP2 shRNA#1-3':
AATTCAAAAAAGTGAAGCTGGAAGCGCATATCTCGAGATATGCGCTTCCAGCTTCACT
hIGF2BP2 shRNA#2-5':
CCGGCTTAACCAGTGCAGAAGTCATCTCGAGATGACTTCTGCACTGGTTAAGTTTTTG
hIGF2BP2 shRNA#2-3':
AATTCAAAAAACTTAACCAGTGCAGAAGTCATCTCGAGATGACTTCTGCACTGGTTAAG
hIGF2BP3 shRNA#1-5':
CCGGGCAGGAATTGACGCTGTATAACTCGAGTTATACAGCGTCAATTCCTGCTTTTTG
hIGF2BP3 shRNA#1-3':
AATTCAAAAAGCAGGAATTGACGCTGTATAACTCGAGTTATACAGCGTCAATTCCTGC
hIGF2BP3 shRNA#2-5':
CCGGCGGTGAATGAACTTCAGAATTCTCGAGAATTCTGAAGTTCATTCACCGTTTTTG
hIGF2BP3 shRNA#2-3':
AATTCAAAAACGGTGAATGAACTTCAGAATTCTCGAGAATTCTGAAGTTCATTCACCG
